# ZMYM2 restricts 53BP1 at DNA double-strand breaks to favor BRCA1 loading and homologous recombination

**DOI:** 10.1093/nar/gkac160

**Published:** 2022-03-07

**Authors:** Doohyung Lee, Katja Apelt, Seong-Ok Lee, Hsin-Ru Chan, Martijn S Luijsterburg, Justin W C Leung, Kyle M Miller

**Affiliations:** Department of Molecular Biosciences, The University of Texas at Austin, Austin, TX 78712, USA; Department of Human Genetics, Leiden University Medical Center, Einthovenweg 20, 2333 ZC Leiden, the Netherlands; Department of Radiation Oncology, College of Medicine, University of Arkansas for Medical Sciences, Little Rock, AR 72205, USA; Department of Molecular Biosciences, The University of Texas at Austin, Austin, TX 78712, USA; Department of Human Genetics, Leiden University Medical Center, Einthovenweg 20, 2333 ZC Leiden, the Netherlands; Department of Radiation Oncology, College of Medicine, University of Arkansas for Medical Sciences, Little Rock, AR 72205, USA; Department of Molecular Biosciences, The University of Texas at Austin, Austin, TX 78712, USA; Livestrong Cancer Institutes, Dell Medical School, The University of Texas at Austin, Austin, TX 78712, USA

## Abstract

An inability to repair DNA double-strand breaks (DSBs) threatens genome integrity and can contribute to human diseases, including cancer. Mammalian cells repair DSBs mainly through homologous recombination (HR) and nonhomologous end-joining (NHEJ). The choice between these pathways is regulated by the interplay between 53BP1 and BRCA1, whereby BRCA1 excludes 53BP1 to promote HR and 53BP1 limits BRCA1 to facilitate NHEJ. Here, we identify the zinc-finger proteins (ZnF), ZMYM2 and ZMYM3, as antagonizers of 53BP1 recruitment that facilitate HR protein recruitment and function at DNA breaks. Mechanistically, we show that ZMYM2 recruitment to DSBs and suppression of break-associated 53BP1 requires the SUMO E3 ligase PIAS4, as well as SUMO binding by ZMYM2. Cells deficient for ZMYM2/3 display genome instability, PARP inhibitor and ionizing radiation sensitivity and reduced HR repair. Importantly, depletion of 53BP1 in ZMYM2/3-deficient cells rescues BRCA1 recruitment to and HR repair of DSBs, suggesting that ZMYM2 and ZMYM3 primarily function to restrict 53BP1 engagement at breaks to favor BRCA1 loading that functions to channel breaks to HR repair. Identification of DNA repair functions for these poorly characterized ZnF proteins may shed light on their unknown contributions to human diseases, where they have been reported to be highly dysregulated, including in several cancers.

## INTRODUCTION

Maintaining genome integrity is fundamental for normal cell and organismal functions. However, endogenous and exogenous agents threaten genome integrity through their ability to induce DNA damage and genome instability. Cells counteract DNA damage by utilizing DNA damage response (DDR) pathways, which choreograph the detection and repair of DNA lesions through the engagement with DDR signaling pathways (1). Among various DNA lesions, DNA double-strand breaks (DSBs) are one of the most cytotoxic as a failure to repair these lesions can result in genetic instability, cell death and/or malignant transformation (2). Two primary pathways are employed in mammalian cells to counteract and repair DSBs: nonhomologous end-joining (NHEJ) and homologous recombination (HR). NHEJ repairs DSBs by ligating the two broken ends together with little to no end processing while HR uses a template to copy and synthesize the damaged region to faithfully repair the break. While HR is suppressed in G1 when cells lack a template for repair, both NHEJ and HR are active in S/G2 phase. NHEJ is highly active in mammalian cells throughout the cell cycle, raising the important question of how DSB repair pathway choice, between NHEJ and HR, is determined.

53BP1 (p53-binding protein 1) is a key regulator of DSB pathway choice. 53BP1 is rapidly recruited to damaged chromatin via multivalent interactions with histone marks including H4K20me2 and H2AK15ub that are catalyzed by SET8-SUV4-20H1/2 and RNF168 respectively (3–6). At DNA breaks, 53BP1 antagonizes the HR factor BRCA1 to facilitate DNA repair by NHEJ. Several other factors have been identified in the 53BP1 DDR axis, including RIF1, which interacts with 53BP1 and recruits REV7 and the Shieldin complex that inhibits DNA end resection, a key step in HR (7–13). In addition, PTIP also interacts with 53BP1 to recruit ARTEMIS and promote classical NHEJ (7,14). While the downstream signal pathway of 53BP1 and its engagement with chromatin marks that regulate repair pathway choice have been heavily studied, how BRCA1 antagonism is regulated by 53BP1 and potentially other factors at break sites to modulate DNA repair path selection remains incompletely understood.

Zinc-finger proteins (ZnFs) represent one of the largest groups of proteins encoded in the human genome and are reported to be involved in various cellular functions including the DDR (15,16). We previously determined that ZMYM3, a member of the myeloproliferative, and mental retardation (MYM)-type ZnF family, plays critical roles in DSB repair, including promoting the association of the HR factor BRCA1 with DSBs (17). In addition to ZMYM3, there are five other mammalian proteins that contain MYM-type zinc finger motifs (18). Here, by performing DSB repair and localization screens for all ZMYM proteins, we identify ZMYM2 as a new DNA damage response factor that constrains 53BP1 chromatin binding and promotes DNA double-strand break repair by BRCA1-dependent homologous recombination. This pathway is governed by SUMOylation as the E3 ligase PIAS4 promotes ZMYM2 recruitment to damage sites and SUMO binding by ZMYM2 mediates restriction of break-associated 53BP1 and efficient loading of BRCA1 at DSBs. Thus, our findings reported here provide mechanistic insights into how DSB repair pathway choice at the level of 53BP1 and BRCA1 is orchestrated by SUMOylation through the ZMYM2 and ZMYM3 effectors of this break-associated modification. As mutations in ZMYM2 and ZMYM3 have been identified in several cancers, these results may reveal cancer-relevant functions of these poorly characterized ZnF proteins.

## MATERIALS AND METHODS

### Cell culture and treatments

Human osteosarcoma U2OS cells and human embryonic kidney HEK293T cells were obtained from ATCC (American Type Culture Collection). U2OS and HEK293T cells were cultured in Dulbecco's modified Eagle's medium (DMEM) with 10% fetal bovine serum (FBS) supplemented with 2 mM l-glutamine, 100 U/ml penicillin, and 100 μg/ml streptomycin (Invitrogen) at 37°C in 5% (v/v) CO_2_. U2OS DSB-reporter cell line (19) was used to monitor the recruitment of BRCA1 and 53BP1 to the FokI-mediated DSBs. 48h post-transfection with the indicated siRNA transfection, mCherry-LacI-FokI fused proteins were induced by adding Shield-1 (Clonetech) and 4-OHT for 4 h and analyzed. U2OS cells were treated with the PARP inhibitor Olaparib as indicated. X-ray irradiation was induced by an X-ray generator (Faxitron X-ray system, RX650). UV irradiation was performed using UV-C source (Philips TUV 9W PL-S).

### Plasmids and cloning

Human ZMYM family genes (ZMYM2, ZMYM3, ZMYM4 and ZMYM6) were amplified by PCR from HEK293T cDNAs. Amplified ZMYM gene products were cloned into a pDONR201 vector using Gateway cloning (Invitrogen) following the manufacturer's protocol. ZMYM1 in pENTR223 vector was purchased from Harvard plasmid (Clone ID:HsCD00365611). ZMYM5 in M29 vector was purchased from (GeneCopoeia, Inc., Cat No. EX-Z6750-M29). ZMYM cDNAs were then sub-cloned into pDEST mammalian expression vector as described previously (17). For ZMYM2, this gene was sub-cloned from pDONR201 into various Gateway destination vectors containing epitope tags as indicated. Deletion mutants of ZMYM2 were created in pDONR201 by PCR-amplifying ZMYM2 cDNA using forward and reverse primers containing complementary sequences to the regions flanking the area to be deleted. SUMO-interacting motif mutant forms of ZMYM2 were generated by site-directed mutagenesis (New England Biolabs) using primers that created alanine substitutions at the indicated amino acid positions.

### Reverse transcription and quantitative PCR (qPCR)

Total RNA was extracted from cells transfected with the indicated siRNAs using the RNeasy kit (Qiagen) with RNAse-Free DNase I (Qiagen) following the manufacturer's protocol. 1 μg of total RNA was reverse-transcribed to synthesize cDNA using superscript III first strand synthesis system (Invitrogen). Quantitative PCR was performed using Fast SYBR™ green master mix (Thermo fisher) on the StepOnePlus Real-time PCR system (Applied Biosystems).

### Primers

List of primers used in this study. ZMYM4 RT-qPCR (Fw #1: 5′-GCTTTCCTTTGCCCATGTG-3′, Rv #1: 5′-CTTCATTTCGTTTCCTCTTGCC-3′, Fw #2: 5′- GAAAGGGTCTACTCAGCTATTCTG-3′, Rv #2: 5′-AACTGGGCACTGATCACATC-3′). ZMYM5 RT-qPCR (Fw #1: 5′- CAAGACTGGAGTAAGACCTTTTAAC-3′, Rv #1: 5′- GGATTTGCTTGATTCTACTGGAAG-3′, Fw #2: 5′-TGGAGTAAGACCTTTTAACCCTG-3′, Rv #2: 5′- CCCCTGGCTGTTTCTGATT-3′). ZMYM6 RT-qPCR (Fw #1: 5′-AGGTGTCCAGGTTTCATGTC-3′, Rv #1: 5′-AGTTTGTTCCTTTTGGCTTGC-3′, Fw #2: 5′- CATATCATGCAAACCCGTCAC-3′, Rv #2: 5′-AGGTGGAGAATCAAGTTCTGC-3′). ZMYM1 Gateway Cloning (Fw: 5′-GGGGACAAGTTTGTACAAAAAAGCAGGCTTCATGAAAGAACCACTTTTAGG-3′, Rv: 5′-GGGGACCACTTTGTACAAGAAAGCTGGGTCTGAGCATGTATTATATTTCTTTC-3′). ZMYM2 Gateway Cloning (Fw: 5′-GGGGACAAGTTTGTACAAAAAAGCAGGCTTCATGGACACAAGTTCAGTGGGAG-3′, Rv: 5′- GGGGACCACTTTGTACAAGAAAGCTGGGTCTTAGTCTGTGTCTTCATCCAG-3′). ZMYM4 Gateway Cloning (Fw: 5′-GGGGACAAGTTTGTACAAAAAAGCAGGCTTCATGGCGGAGAGAGAGGTGGAG-3′, Rv: 5′-GGGGACCACTTTGTACAAGAAAGCTGGGTCTTAATCTGATAATTCAACATCAG-3′). ZMYM6 Gateway Cloning (Fw: 5′-GGGGACAAGTTTGTACAAAAAAGCAGGCTTCATGAAAGAACCTTTGGATGG-3′, Rv: 5′-GGGGACCACTTTGTACAAGAAAGCTGGGTCCTACTCTTTCTCCTTCACTAAT-3′). ZMYM2 TRASH deletion Cloning (Fw: 5′-GCTCACCTCTTTTGTTCTAC-3′, Rv: 5′-GAAAATCTGTTTAGGAAGTGAG-3′). ZMYM2 ZnF deletion Cloning (Fw: 5′- CTACTAAACCAGTTAAAGTCACTCCCAACATGACAACTCAGAAAGG-3′, Rv: 5′-CCTTTCTGAGTTGTCATGTTGGGAGTGACTTTAACTGGTTTAGTAG-3′). ZMYM2 SIM1 substitution mutant (Fw: 5′-GCAGCGGAACCTGTACAACCTCCC-3′, Rv: 5′-GGCAGCATCATCATCATCTTCCACTG-3′). ZMYM2 SIM2 substitution mutant (Fw: 5′-GCAGCGGATGATGAAGAGGACATG-3′, Rv: 5′-GGCAGCCTCACTTACACTTCCCTTC-3′). ZMYM2 SIM3 substitution mutant (Fw: 5′-GCAGCGGATGGTCAACAGAAAAGATTTTG-3′, Rv: 5′-GGCAGCATTATTTCCAGCACCTTTAC-3′).

### Generation of knockout cell lines

ZMYM2 knockout (KO) U2OS clones were established using CRISPR/Cas9 gene editing. The single-guide RNA (sgRNA) sequences used for ZMYM2 was 5′- GCAACTAGTCTCACGAATGT-3′. ZMYM2 gRNA was subcloned into pSpCas9 (BB)-2A-Puro (PX459, Addgene #48139) and then transfected into U2OS cells using Lipofectamine 2000 (Invitrogen) according to manufacturer's instructions. Single clones were selected by limited dilution, and protein levels were screened by western blotting (WB) and immunofluorescence (IF) using ZMYM2 antibodies (Bethyl Laboratories #A301-709A 1:1000, Abcam #ab106624 1:1000, and Sigma #HPA031765 1:100, respectively). To generate XPC knockouts, U2OS(FRT) cells were co-transfected with pLV-U6g-PPB encoding an XPC guide RNA from the LUMC / Sigma-Aldrich sgRNA library (5-TGGGGGTTTCTCATCTTCAAAGG-3) together with an expression vector encoding Cas9-2A-GFP (pX458; Addgene #48138) using lipofectamine 2000 (Invitrogen). Transfected U2OS (FRT) cells were selected on puromycin (1 μg/ml) for three days, plated at low density after which individual clones were isolated. Knockout clones were verified by WB analysis using XPC antibodies (Novus Biologicals #NB100-58801 1:1000).

### Plasmids and siRNA transfection

Mammalian expression vectors were transiently transfected into the indicated cell lines using Polyethylenimine (Polysciences), Lipofectamine 2000 (Invitrogen) or Fugene HD (Promega) following the manufacturer's instruction. siRNA transfections were performed with Lipofectamine RNAiMAX (Invitrogen) according to the manufacturer's protocol. Here are the specific siRNA sequences used for targeting the indicated gene products; ZMYM1 (ON-TARTGETplus siZMYM1 SMARTpool L-007066-02-0005); ZMYM2 (ON-TARTGETplus siZMYM2 SMARTpool L-021348-00-0005); ZMYM3 (ON-TARTGETplus siZMYM3 SMARTpool L-019933-00-0005); ZMYM4 (ON-TARTGETplus siZMYM4 SMARTpool L-019932-02-0005); ZMYM5 (ON-TARTGETplus siZMYM5 SMARTpool L-020648-02-0005); ZMYM6 (ON-TARTGETplus siZMYM5 SMARTpool L-016304-02-0005); BRCA1 (5′-CAGCAGUUUAUUACUCACUAAUU-3′); LigaseIV (5′-AGGAAGUAUUCUCAGGAAUUAUU-3′ (20)); 53BP1 (#1; 5′-CAGGACAGUCUUUCCACGA-3′ (21), #2; 5′-GAACAGAAGUAGAAAGAAAUU-3′); PIAS1 (FlexiTube PIAS1 siRNA #SI00113974); PIAS4 (FlexiTube PIAS4 siRNA # SI00684439); and RNF168 (5′-GACACUUUCUCCACAGAUAUU-3′ (22)); and MDC1 (5′-GUUGUAACUGAAAUCCAGC-3′ (23)). SMARTpool siRNAs and FlexiTube siRNAs were purchased from Dharmacon and QIAGEN respectively. Other siRNAs were purchased from Sigma-Aldrich. siRNA knockdown efficiencies were validated by WB and/or RT-qPCR.

### Protein extraction and western blotting

Whole-cell lysates were obtained from cells as previously described (24). Briefly, cells were washed with PBS, collected with radioimmunoprecipitation assay (RIPA) lysis buffer containing 1% Nonidet P-40, 0.5% sodium deoxycholate, and 0.1% sodium dodecyl sulfate. For chromatin fraction, cells were washed twice with PBS and treated with CSK buffer for 5 min on ice followed by washing with PBS three times. Cell lysates were collected with RIPA lysis buffer. WB samples were resolved by SDS-PAGE and analyzed by standard WB protocols. Signals of the western blots were detected by a standard chemiluminescence reagent (Cytivia) and analyzed using a ChemiDoc system (Bio-Rad Laboratories) using indicated antibodies.

### Antibodies

Primary antibodies used in this study were ZMYM1 (Novus Biologicals, NBP1-76523), ZMYM2 (Bethyl laboratories, A301-709A), ZMYM2 (Abcam, ab106624), ZMYM2 (Sigma, HPA031765), ZMYM3 (Bethyl laboratories, A300-200A), ZMYM3 (Bethyl laboratories, A300-264A-M), 53BP1 (Novus Biologicals, NB100-304), BRCA1 (Santa Cruz, sc-6954), RAP80 (Bethyl laboratories, A300-763A), ABRA1 (Abcam, ab139191), MDC1 (Abcam, ab11169), DNA-Pkcs (Cell signaling, 4602), Phospho S2056 DNA-Pkcs (Abcam, ab18192), ATM (Cell Signaling, 2873S), Phospho S1981 ATM (Abcam, ab81292), Chk2 (Cell Signaling, 2662S), Phospho T68 Chk2 (Cell Signaling, 2661S), H2AX (Cell Signaling, 2595S), Phospho S139 H2AX (Cell Signaling, 9718S), Phospho S139 H2AX (Millipore, 05-636), Phospho S10 H3 (Cell Signaling, 3377S), Flag (Sigma Aldrich, F1804), GFP (Abcam, ab290), PIAS1 (Abcam, ab32219), PIAS4 (Abcam, ab58416), CPD (Cosmo Bio CAC-NM-DND-001) and β-Tubulin (Abcam, ab6046). For western blotting analysis, HRP-conjugated anti-mouse and rabbit IgG antibodies (Cell Signaling, 7074 and 7076) were used. For IF or Flow cytometry, Alexa Fluor 488 goat anti-rabbit IgG (Invitrogen, A11034), Alexa Fluor 594 goat anti-rabbit IgG (Invitrogen, A11037), Alexa Fluor 647 goat anti-rabbit IgG (Invitrogen, A21245), Alexa Fluor 488 goat anti-mouse IgG (Invitrogen, A11029), Alexa Fluor 594 goat anti-mouse IgG (Invitrogen,A11032), and Alexa Fluor 647 goat anti-mouse IgG (Invitrogen, A21236) were used.

### Immunoprecipitation analysis

Cells were collected and lysed with NETN lysis buffer (150 mM NaCl, 0.5 mM EDTA, 20 mM Tris–HCl at pH7.5, 0.5% Nonidet P-40) containing 10 mM MgCl_2_, protease inhibitor (Roche), and TurboNuclease (Accelagen). For precipitation of GFP or SFB-tagged proteins, cell lysates were incubated with GFP-Trap magnetic agarose (Chromotek) or streptavidin beads (GE Healthcare) overnight at 4°C respectively. Immunocomplexes were washed with NETN buffer 3× at 4°C and eluted by boiling in 2× sample loading buffer (100 mM Tris–HCl at pH6.8, 4% SDS, 20% glycerol and 200 mM β-mercaptoethanol is added just before the buffer is used) at 95°C. Samples were resolved by SDS-PAGE and immunoblotted with antibodies as indicated.

### HR and NHEJ assay

DSB repair efficiency was determined using cell-based reporter systems as described (17). DR-GFP U2OS and EJ-5 U2OS cells were seeded in six-well plates followed by transfection with the indicated siRNAs (1 μl of 20 μM stock/well) 24 h after seeding. After siRNA transfection (24 h), cells were transfected with a I-SceI expressing vector (pCAG-I-SceI) or control vector (pCAG-empty). Cells were collected 72 h post-siRNA transfection and GFP positive cells were detected using a BD Accuri flow cytometer (BD biosciences). Results were normalized to siRNA control cells transfected with I-SceI expressing vector.

### Immunofluorescence and confocal microscopy

Immunofluorescence (IF) was performed as described previously (25). Briefly, cells were cultured on pre-coated glass coverslips for IF or glass-bottom plates for live-cell imaging containing normal cell culture medium before analysis. Cells were washed with PBS three times and pre-extracted with CSK buffer (10 mM PIPES pH 6.8, 100 mM NaCl, 300 mM sucrose, 3 mM MgCl_2_, 1 mM EGTA, 0.5% Triton X-100) for 5 min on ice followed by 15 min fixation with 2% paraformaldehyde in PBS at room temperature. Samples were washed with PBS three times and then incubated with the indicated antibodies overnight at 4°C. On the following day, samples were washed with PBS three times, and incubated with secondary antibodies for 1 h at room temperature. Samples were then mounted with VECTASHIELD mounting solution containing DAPI (Vector Labs). Samples were visualized using an inverted FV3000 scanning confocal microscope (Olympus). Z-stacked images were obtained for images and focus counting, which was performed with FW31S software.

### Clonogenic cell survival assays

Cells were seeded at a density of 500 cells per well in six-well plates in triplicate. Cells were exposed to increasing doses of UV-C light or IR or treated for 1 day (24h) with the indicated concentrations of Olaparib. Cells were subsequently incubated for 10–14 days until colonies were formed. Plates were washed with PBS twice before colonies were fixed and stained with 0.5% crystal violet in 20% ethanol. Colonies were manually counted and normalized to untreated cells.

### MTT assays

Cells were seeds at 2000 cells/well in 96 well plates. After 24 h, cells were treated with either cisplatin, mitomycin C, olaparib or DMSO at the indicated concentrations for an additional 72 h. Cells were then treated with MTT [3-(4,5-dimethylthiazol-2-yl)-2,5-diphenyltetrazolium bromide, 5 mg/ml in PBS] (Sigma) solution for 4 h at 37°C. Formazan were resolved with DMSO. The absorbance of samples was read at 565 nm using an infinite M1000Pro plate reader (TECAN).

### Live-cell imaging

Laser microirradiation analysis was performed using an inverted FV3000 scanning confocal microscope (Olympus) as previously described (20,26). Cells were seeded onto glass coverslips or glass-bottomed dishes (Willco Wells) and incubated with 10 μM BrdU for 24 h prior to laser microirradiation. For live-cell imaging of GFP-tagged protein damage recruitment studies, cells were transiently transfected with GFP-tagged proteins and following 1–2 days for proteins to express, samples were subjected to laser-induced damage with a fixed-wavelength 405 nm laser using a 60× objective at 60% power on an inverted FV3000 scanning confocal microscope (Olympus). Live images were captured in 30s intervals. Fluorescence intensity of the GFP-tagged protein in the damaged region was normalized with an equivalent undamaged area in the same cell.

### UV-A laser micro-irradiation

Cells were grown on 18-mm glass coverslips and transfected with 500 ng plasmid DNA encoding GFP-ZMYM2 or GFP-ZMYM3 using Lipofectamine 2000 overnight, according the manufacturer's instructions. The next day cells were sensitized with 15 μM 5′-bromo-2-deoxyuridine (BrdU) for 24 h to generate DSB and incubated with 2 μg/ml doxycycline to induce expression of GFP-tagged proteins. At 24 h after doxycycline incubation, cells were placed in a Chamlide CMB magnetic chamber in which growth medium was replaced by CO_2_-independent Leibovitz's L15 medium (Thermo Fisher). UV-A laser tracks were generated using a diode-pumped solid state 355 nm Yttrium Aluminum Garnet laser (average power 14 mW, repetition rate up to 200 Hz). The laser was integrated in a UGA-42-Caliburn/2L Spot Illumination system (Rapp OptoElectronic). Microirradiation was combined with live-cell imaging in an environmental chamber set to 37°C on a widefield fluorescence Zeiss Axio Observer 7 microscope, using a Plan-Neofluar 63x (1.25 NA) oil-immersion objective. The laser system is coupled to the microscope via a triggerbox and a neutral density (ND-1) filter blocks 90% of the laser light. A HXP 120 V metal-halide lamp was used for excitation. Images were acquired in Zeiss ZEN and quantified in ImageJ.

### Mitotic index and cell cycle

Mitotic cell analysis was performed as described (17). Cells were treated with ionization radiation with the indicated dose and collected. Cells were trypsinized and fixed in 80% ethanol overnight at 4°C. For the mitotic index, cells were stained with H3 S10-P (Cell Signaling #3377) for 2 h followed by Alexa fluor 488 for 2 h at room temperature. Cell cycle was analyzed by staining with 4 μg/ml propidium iodide followed by treatment with 2 μg/ml RNase A for 30 min at room temperature. Cells were analyzed by flow cytometry, and data were processed with FlowJo 10 software.

### Cell proliferation

Cell proliferation was measured by Incucyte^®^ (Satorious). Cells were seeded 2000 cells/well in 96-well plates, and cell proliferation rate was monitored by the percentage of confluent cells over the indicated time.

### Tandem affinity purification (TAP) and mass spectrometry (MS) analysis

IPed samples were prepared as previously described (27). Briefly, HEK293T cells were transiently transfected with SFB-tagged ZMYM2. Cells were extracted with NETN buffer for 30 min at 4°C. Cell lysates were centrifuged, and the supernatants were collected as the soluble fraction. The pellets were digested with NETN buffer with Turbo-Nuclease and 1 mM MgCl_2_ for 1 h at 4°C and collected as the chromatin fraction. The both fractions were incubated with streptavidin sepharose bead (GE healthcare) for 1 h at 4°C. The beads were washed twice with NETN buffer and eluted with 2 mM biotin at 4°C. The eluted supernatants were incubated with S-protein beads (EMD Millipore) overnight at 4°C and washed three times with NETN buffer. The protein mixtures were eluted by boiling with 1× sample buffer. The eluted complex was subjected to SDS-PAGE and MS analysis.

### Unscheduled DNA synthesis (UDS)

∼180,000 cells were seeded on 18-mm glass coverslips in 12-wells plates in DMEM. The following day, the medium was replaced with DMEM. After 24 h, cells were locally irradiated through a 5 μm filter with 30 J/m^2^ UV-C. Cells were subsequently pulse-labeled with 20 μM 5-ethynyl deoxy-uridine (EdU; VWR) and 1 μM FuDR (Sigma Aldrich) for 1 h. After labeling, cells were chased with 10 μM thymidine in DMEM without supplements for 30 min and fixed for 15 min with 3.7% formaldehyde in PBS. Cells were permeabilized for 20 min in PBS with 0.5% Triton-X100 and blocked in 3% bovine serum albumin (BSA, Thermo Fisher) in PBS. The incorporated EdU was coupled to Attoazide Alexa Fluor 647 using Click-iT chemistry according to the manufacturer's instructions (Invitrogen). After coupling, the cells were post-fixed with 2% formaldehyde for 10 min and subsequently blocked with 100 mM Glycine. DNA was denatured with 0.5 M NaOH for 5 min, followed by blocking with 10% BSA (Thermo Fisher) for 15 min. Next, the cells were incubated with an antibody against CPDs (host: mouse; Cosmo Bio CAC-NM-DND-001; 1:1000) for 2 h, followed by secondary antibodies (host: goat, anti-mouse IgG (H + L) CF770; Biotium, VWR #20077; 1:10,000) 1 h, and DAPI for 5 min. Cells were mounted in Polymount (Brunschwig). UDS experiments were performed in duplicate and repeated three times.

## RESULTS

### ZMYM2 is a DNA damage response factor

**Figure 1.**
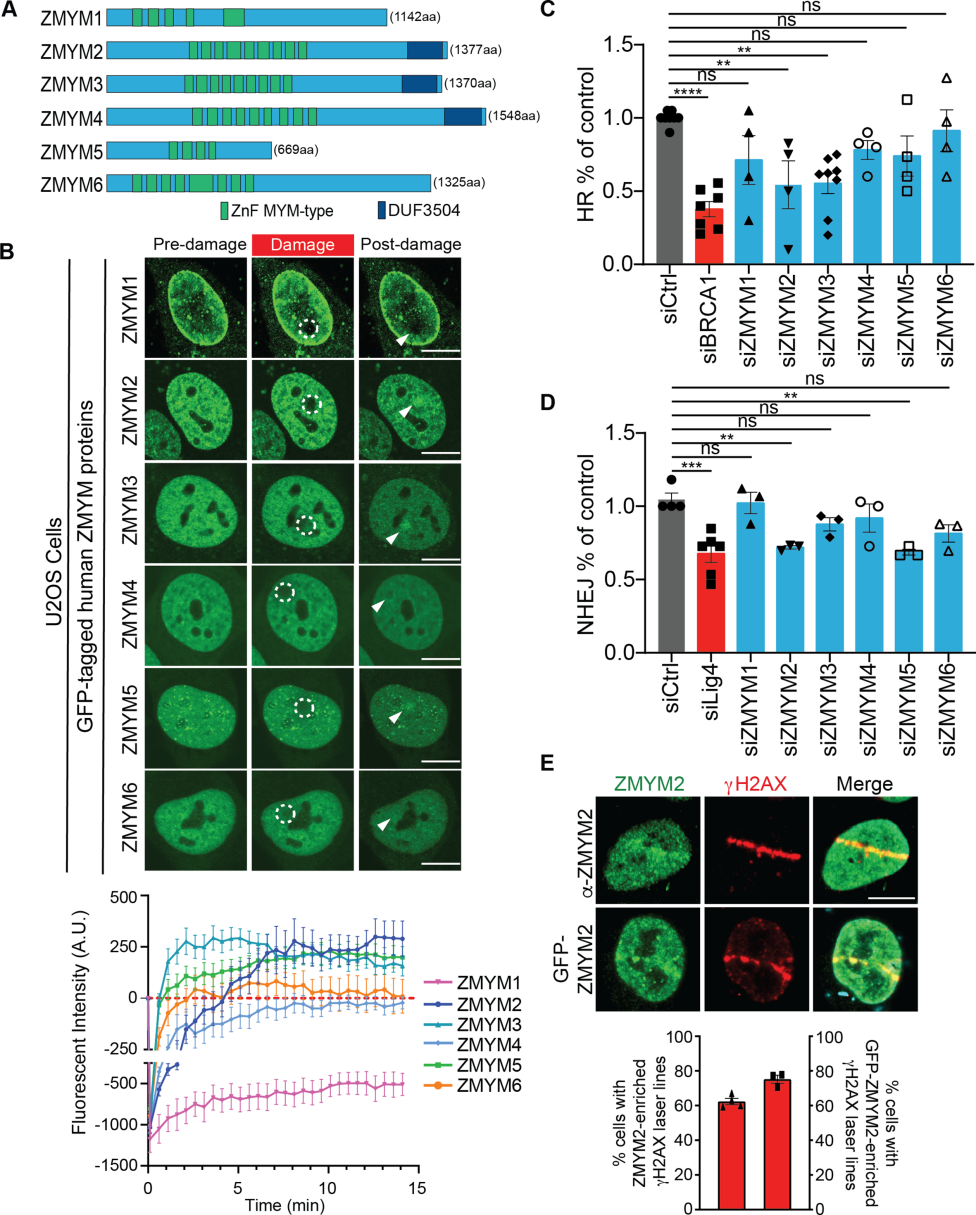
ZMYM2 localizes to DNA lesions and promotes DNA double-strand break repair. (**A**) Schematic illustration of the human Zinc Finger (ZnF) MYM-type protein family. Total amino acids for each full-length protein are indicated. Locations of the myeloproliferative and mental retardation-type zinc fingers (ZnF MYM-type, Green) and domain of unknown function (DUF3504, Dark blue) are shown. (**B**) Screen for laser-damage recruitment of human ZMYM proteins. U2OS cells expressing GFP-tagged ZMYM proteins were analyzed 15 min post-damage by fluorescence confocal live-cell microscopy. Dotted white circles indicate damaged region and white arrowhead points to post-damage region. Lower panel; Quantification of laser damage recruitment. Data represent mean ± S.E.M., *N* ≥ 9 cells for ZMYM3 expressing cells and ≥12 cells for all other ZMYM protein expressing cells compiled from >2 independent biological replicates. (**C**) Homologous recombination (HR) efficiency in ZMYM-deficient cells. HR levels were obtained in DR-GFP U2OS cells transfected with siRNAs targeting individual ZMYM genes. BRCA1 acts as a positive control. Data represent mean ± S.E.M., *n* ≥ 3. (**D**) Nonhomologous end-joining (NHEJ) analysis. NHEJ efficiencies were obtained in EJ5 U2OS cells transfected with siRNAs targeting individual ZMYM genes. Ligase IV acts as a positive control. Data represent mean ± S.E.M., *n* ≥ 3, (**P < 0.01, ***P < 0.001, ****P < 0.0001, one-way ANOVA test for (C, D); non-targeting siCtrl was used as a negative control). (**E**) ZMYM2 recruitment to laser damage. (Top) WT U2OS cells and (Bottom) WT U2OS cells transiently expressing GFP-tagged ZMYM2 were damaged and analyzed at 1-hour post-damage by fluorescence confocal live-cell microscopy. Endogenous ZMYM2 protein was detected by IF using a ZMYM2 specific antibody that was validated in Supplementary Figure S1D. Data represent mean ± S.E.M., *n* ≥ 20 cells per each independent experiment, *n* = 3, All scale bars, 10 μm.

There are six human (MYM)-type ZnF family members, which each possess a conserved array of MYM-type ZnF domains that range from four to nine individual ZnFs (Figure 1A). In addition, ZMYM2, ZMYM3 and ZMYM4 contain a C-terminal domain of unknown function (DUF3504). We previously identified a member of this protein family, ZMYM3, as a DDR factor (17). Based on these findings, we hypothesized that other MYM-type ZnF proteins may similarly localize and act at DNA damage sites to promote DNA repair. To explore this idea, we tested all the remaining five ZMYM family members for their ability to associate with DNA lesions using laser-microirradiation and live-cell imaging by confocal microscopy. Using GFP-tagged ZMYM proteins in this assay, we observed that ZMYM2 and ZMYM5 were recruited to DNA damage sites whereas, ZMYM1, ZMYM4 and ZMYM6 were not (Figure 1B; note a fluorescence intensity above zero indicates an accumulation of the GFP-tagged proteins at damaged compared to undamaged regions; see methods for details). As expected, ZMYM3 was also recruited to damage sites as previously shown (17). Interestingly, we found that ZMYM1 fluorescence signal did not recover in DNA damage regions, suggesting that this factor may be highly immobile in cells and/or actively displaced from DNA lesion sites (Figure 1B). These results suggest that other members of the (MYM)-type ZnF family in addition to ZMYM3 are involved in the DDR.

Given that recruitment to DNA damage is often a characteristic of proteins involved in DNA repair, we next ascertained the requirement of ZMYM proteins in DSB repair using cell-based assays for both DSB repair by homologous recombination and nonhomologous end-joining (i.e. DR-GFP HR and EJ5-NHEJ; (28,29)). Consistent with our DNA damage recruitment analysis, U2OS cells depleted of ZMYM2 or ZMYM5 by siRNA exhibited reduced DSB repair compared to siCtrl treated U2OS cells (Figure 1C and D; siBRCA1 and siLig4 act as positive controls for HR and NHEJ respectively). Successful knockdown of ZMYM1-6 by siRNA was confirmed by either western blotting or RT-qPCR (Supplementary Figure S1A and B), depending on available antibodies. Results from these DSB repair assays revealed that ZMYM2-deficiency reduced both HR and NHEJ repair efficiency while cells depleted of ZMYM5 displayed a defect only in NHEJ. Further characterization of the DNA damage recruitment dynamics of GFP-tagged ZMYM2 revealed the colocalization of this protein with the DSB marker γH2AX, which was observable even 1 h post-damage in over 70% of damaged cells (Figure 1E). These results suggested that ZMYM2 may function as a new factor involved in DSB repair.

To build upon these observations and further study the potential involvement of ZMYM2 in genome integrity and the DDR, we generated ZMYM2 knockout (KO) U2OS cell lines using CRISPR-Cas9 gene-editing. Western blotting analysis confirmed ZMYM2 protein loss in several clones (Supplementary Figure S1C). We also confirmed the loss of ZMYM2 in these clones by immunofluorescence (IF) analysis (Supplementary Figure S1D), which additionally validated the ZMYM2 antibody for recognizing this protein specifically using either western blotting or IF approaches. We also confirmed by western blotting that the loss of either ZMYM2 or ZMYM3 did not alter the protein level of the other ZMYM protein (Supplementary Figure S1C). This result ruled out the potential for these ZMYM proteins to co-regulate the expression of each other, providing additional evidence that the DDR defects associated with the loss of each factor in either ZMYM2 or ZMYM3 KO cells is related to the deficiency of the specific gene being edited (Supplementary Figure S1C) (17). Further analysis also revealed that the loss of ZMYM2 in U2OS cells did not alter cell cycle distribution or proliferation of ZMYM2 KO cells compared to parental U2OS cells (Supplementary Figure S1E and F).

The availability of a specific ZMYM2 antibody that worked in IF prompted us to examine the recruitment of endogenous ZMYM2 to DSBs, to rule out the potential impact of overexpression and GFP-tagging on the behavior of ZMYM2 in DNA break recruitment assays. Using the same experimental system to generate laser damage, we observed recruitment of endogenous ZMYM2 to these breaks using a specific ZMYM2 antibody against the endogenously expressed protein (Figure 1E). We further validated the recruitment of endogenous ZMYM2 to nuclease-generated DSBs using the mCherry-LacI-FokI U2OS-DSB-reporter system (19). Indeed, expression of mCherry-LacI-FokI resulted in the focal accumulation of FokI that led to the generation of γH2AX labelled DSBs, with over 80% of FokI-γH2AX foci displaying accumulation of endogenous ZMYM2 (Supplementary Figure S1G; quantified in H). Thus, these results identify ZMYM2 as a DSB-associated factor and provide strong evidence for the involvement of ZMYM2 in DSB repair.

Out of all the MYM-type ZnF factors analyzed, only ZMYM2 was required for both NHEJ and HR (Figure 1C and D). To identify how ZMYM2 may be involved in NHEJ, we performed tandem affinity purification (TAP) of SFB-tagged ZMYM2 to identify protein interactors. Mass spectrometry analysis of immunopurified ZMYM2 protein complexes revealed 10 high confidence putative interactors that were identified in both of two-independent experiments from chromatin fractions with greater than 9 unique peptides (Supplementary Figure S2A). Of the proteins we identified, the majority of these factors have been previously reported and validated as interacting partners of ZMYM2, including ZMYM3 and members of the LSD1-CoREST-HDAC complex (17,^30^,^31^). Interestingly, we identified the DNA-dependent protein kinase DNA-PK (PRKDC) as a high confidence ZMYM2 interactor, a finding of particular interest given the central role that DNA-PK plays in NHEJ (32). Additional IP western blotting analysis of ZMYM2 co-immunoprecipitated proteins confirmed these results (Supplementary Figure S2B; HDAC2 acts as a positive control for a ZMYM2 interactor). Using ZMYM2 KO cells, we analyzed DNA damage signaling in response to IR using DNA-PK auto-phosphorylation at serine 2056, a PTM induced by DNA-PK activation and commonly used as a proxy for DNA-PK and NHEJ activity in cells (33). Using both IF and western blotting analysis, we observed reduced DNA-PK S2056 phosphorylation after IR damage in ZMYM2 KO cells compared to parental U2OS cells (Supplementary Figure S2C and D). These data provide additional evidence pointing to the involvement of ZMYM2 in promoting efficient NHEJ repair in cells.

ZMYM2 (also known as ZnF198) is fused with the FGFR1 receptor tyrosine kinase in bone marrow cells from patients with an atypical myeloproliferative disease and its expression as a fusion with FGFR1 renders cells sensitive to UV irradiation (34). Given that laser microirradiation using a continuous wavelength 405 nm laser can lead to UV damage, in addition to DSBs (35), we considered whether ZMYM2 may also participate in UV damage responses in addition to DSB repair, including nucleotide excision repair (NER). To further assess the potential involvement of either ZMYM2 or ZMYM3 in UV damage repair, we measured 5-ethynyl-2′-deoxyuridine (EdU) incorporation as a measure for NER activity at sites of local UV damage marked by cyclobutane pyrimidine dimer (CPD) staining in U2OS cells (36,37). Cells lacking either ZMYM2 or ZMYM3 did not display any defect in repair synthesis at UV lesions, which correlates with an ability to incorporate EdU at the site of UV radiation (Supplementary Figure S3A and B) (38). In contrast, XPC KO cells, which are defective in global genome NER, failed to incorporate EdU at the site of UV damage due to defective repair synthesis. Consistent with these results, ZMYM2 or ZMYM3 KO cells were not sensitive to UV irradiation compared to WT cells, which is in stark contrast to XPC KO cells, which displayed strong sensitivity to UV irradiation compared to WT cells (Supplementary Figure S3C). Taken together, these results ruled out a function for ZMYM2 or ZMYM3 in UV repair and instead suggested that ZMYM2 and ZMYM3 function more specifically in the repair of DSBs.

**Figure 2.**
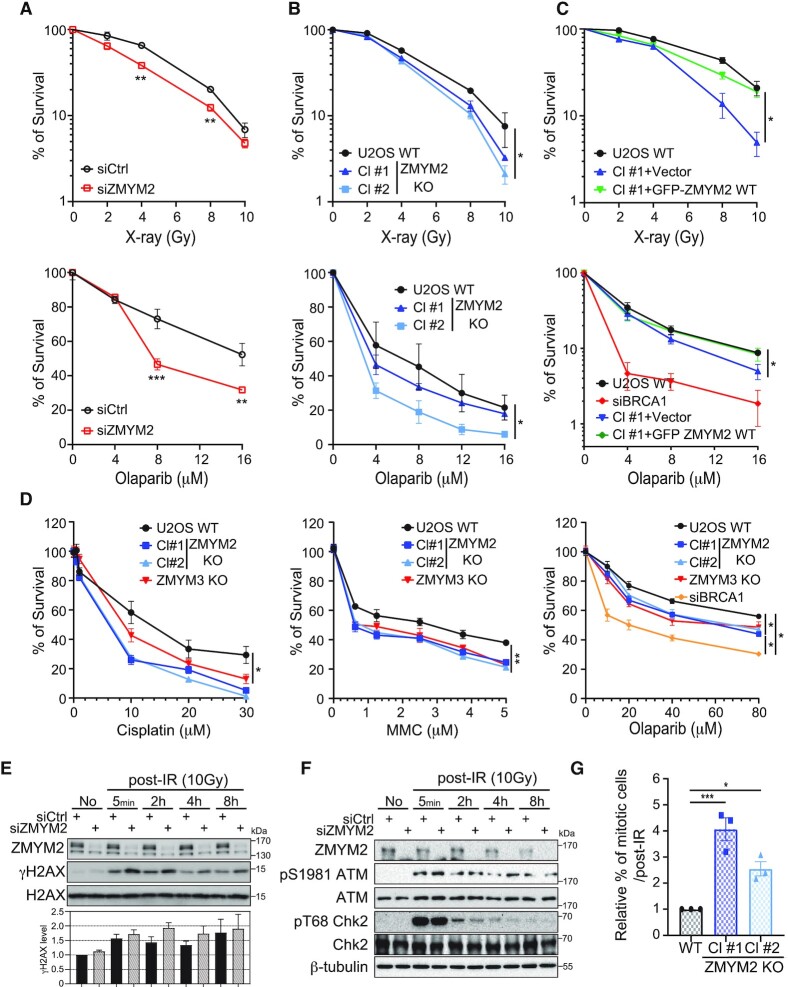
ZMYM2-deficiency results in defective responses and survival to DNA damage and PARP inhibitors. (**A, B**) ZMYM2-deficient cells are sensitive to ionizing radiation (IR; top panel) and PARP inhibitors (PARPi; bottom panel). U2OS cells either treated with control or ZMYM2 siRNAs or depleted of ZMYM2 by CRISPR-Cas9 gene editing were challenged with IR or PARP inhibitors as indicated and analyzed by colony formation assays. Data represent mean ± S.E.M., *n* ≥ 3 (**P < 0.01, ***P < 0.001; multiple unpaired *t*-test for (A), *P < 0.05; two-tailed paired *t*-test for (B)). (**C**) Complementation analysis of ZMYM2 KO cells. ZMYM2 KO cells with empty vector or ZMYM2 WT were analyzed as in (B), BRCA1 siRNA transfected cells were used as positive control. Data represent mean ± S.E.M., *n* = 3, (*P < 0.05; two-tailed paired *t*-test). (**D**) ZMYM2 or ZMYM3 depletion in U2OS cells results in sensitivity to DNA crosslink agents and PARP inhibitor. ZMYM2 and ZMYM3 KO cells were treated with Cisplatin, Mitomycin C and Olaparib with the indicated dosages. Cell survival was measured by MTT assay. Data represent mean ± S.E.M., *n* ≥ 3 (*P < 0.05, **P < 0.002; two-tailed paired *t*-test). (**E, F**) ZMYM2-deficient cells exhibit defects in DNA damage signaling and checkpoint activation following IR-treatment. siCtrl and siZMYM2 U2OS cells were treated with IR and analyzed by western blotting with the indicated antibodies. (**G**) Parental WT and ZMYM2 KO U2OS cells were damaged with IR (5Gy, 6hr) followed by FACS analysis with anti-H3 pS10 and propidium iodide to identify mitotic cells. Data represent mean ± S.E.M., *n* = 3 (*P < 0.05, ***P < 0.001, one-way ANOVA test).

To further explore the functional importance and involvement of ZMYM2 in DSB repair, we investigated the cellular consequences of depleting ZMYM2 in human cancer cells. ZMYM2-deficient cells, by either siRNA-depletion or CRISPR-Cas9 gene editing, reduced the ability of cells to survive DSBs induced by ionizing radiation (IR) compared to wild-type (WT) control cells (Figure 2A and B; ZMYM2 protein loss shown in Figure 2E and Supplementary Figure S1C and D). Consistent with a role for ZMYM2 in homology-directed repair of DSBs, clonogenic cell survival analysis showed that ZMYM2-deficient cells were also sensitive to PARP inhibitor (PARPi: Olaparib; Figure 2A and B). Complementation of ZMYM2 KO cells with WT ZMYM2 was able to rescue the cellular sensitivity of these cells to IR and PARPi, ruling out any potential off-target effects of gene-editing in these cells and demonstrating that these effects are due to the specific loss of ZMYM2 (Figure 2C; BRCA1-deficient cells act as positive control). These results are in agreement with the well-established sensitivity of HR-defective cells to PARPi, for example BRCA1-deficiency or cells lacking ZMYM3 as we reported previously (17). Consistent with ZMYM2 and ZMYM3 involvement in HR, ZMYM2 and ZMYM3-deficient cells were also sensitive to the DNA crosslinking agents, cisplatin and mitomycin C (MMC) as determined by MTT assay (Figure 2D). We also observed comparable results with colony survival and MTT assays for PARPi sensitivity of ZMYM2- and ZMYM3-deficient cells, further supporting the involvement of these ZnF proteins in cellular responses to DNA damage (Figure 2D). We similarly observed ZMYM2-deficient cells also displayed defects in DNA damage signaling, including a mild but reproducible persistence of total γH2AX following IR-treatment compared to control cells (Figure 2E). ATM activation itself, including CHK2 phosphorylation, were similar in ZMYM2-deficient cells compared to control cells, suggesting that activation of DNA damage signaling following DSB-induction was not overtly altered in ZMYM2-deficient cells (Figure 2F). Upon DNA damage, cell cycle checkpoints also participate in the DDR by arresting the cell cycle to allow for DNA repair reactions to occur in a coordinated manner with the cell cycle stage. Following IR-induced DNA damage, we observed that ZMYM2 KO cells contained an increased number of cells in mitosis compared to WT cells, suggesting an inability of these cells to properly mount a G2 arrest following DNA damage (Figure 2G). This phenotype also occurs in ZMYM3-deficient cells and is well-documented in other chromatin DSB response factors including H2AX and MDC1 (17,39,40). Collectively, these data identify ZMYM2 as a new factor involved in the cellular response to DNA damage.

### DNA damage recruitment of ZMYM2 requires SUMOylation

**Figure 3.**
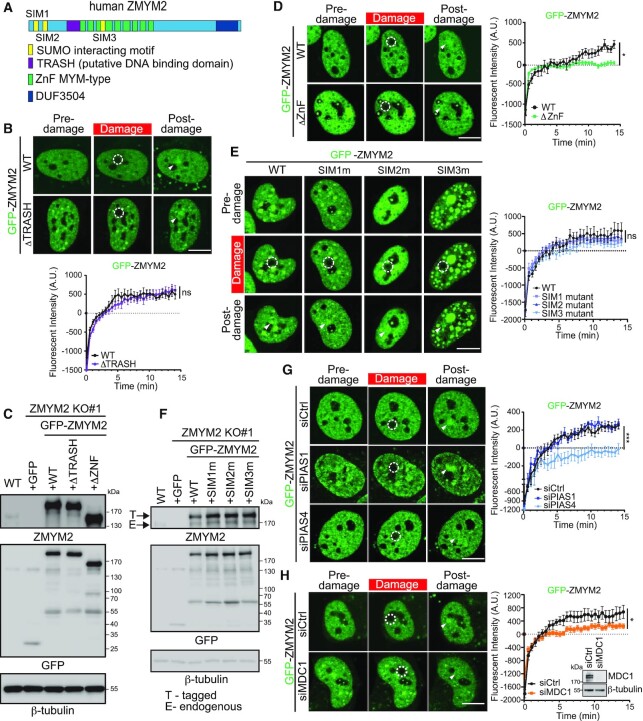
ZMYM2 DNA damage localization requires its ZnF, the E3 SUMO ligase PIAS4 and MDC1. (**A**) Schematic illustration of ZMYM2 domains. SUMO interacting (SIM, yellow), putative DNA binding TRASH (purple), ZnF MYM-type (green) and DUF (dark blue) domains. (**B**) The TRASH domain of ZMYM2 is dispensable for DNA damage recruitment. Experiments were performed as in Figure 1B with GFP-tagged ZMYM2 and TRASH deletion mutant. Data represent mean ± S.E.M., *n* ≥ 8 cells for WT and *n* ≥ 18 cells for ΔTRASH mutant (ns; not significant, two-tailed unpaired *t*-test). (**C**) Protein expression of ZMYM2 WT and mutants was similar. ZMYM2 KO cells were transiently transfected with GFP-tagged ZMYM2 WT, ΔTRASH, or ΔZnF, the expression levels were analyzed using western blotting with the indicated antibodies. (**D**) Recruitment of GFP-tagged ZMYM2 requires the ZnF domain. WT and ZnF domain deletion mutant of GFP-ZMYM2 were transiently expressed in U2OS cells and analyzed as in (B). Data represent mean ± S.E.M., *n* ≥ 7 cells per condition (*P < 0.05, two-tailed unpaired *t*-test). (**E**) SIM mutants are dispensable for damage localization of ZMYM2. Cells expressing GFP-ZMYM2 WT or SIM mutants were analyzed 15 min post-damage by live-cell confocal fluorescence microscopy as in (B). Data represent mean ± S.E.M., *n* ≥ 9 cells (ns; not significant, one-way ANOVA test). (**F**) ZMYM2 WT and SIM mutants expressed similarly. Protein expression was analyzed as in (C). (**G**) The SUMO E3 ligase PIAS4 promotes ZMYM2 damage localization. U2OS cells were treated with the indicated siRNAs and transiently expressed GFP-ZMYM2 was analyzed for damage localization as in (B). Data represent mean ± S.E.M., *n* ≥ 9 cells analyzed per condition, Scale bar = 10 μm, (***P < 0.001, one-way ANOVA test). (**H**) MDC1 is required for ZMYM2 accumulation at DNA lesions. The recruitment of GFP-ZMYM2 was analyzed as in (G). Data represent mean ± S.E.M., *n* ≥ 10 cells per condition (*P < 0.05, two-tailed unpaired *t*-test). All scale bars are 10 μm and dotted white circles indicate damaged regions and white arrowhead points to post-damage region.

While our data identified ZMYM2 as a localizer to DNA damage sites, we next set out to identify the molecular interactions that governed the association of ZMYM2 with DNA lesion sites. In addition to MYM-type ZnFs and a DUF domain, ZMYM2 also contains a putative DNA-binding TRASH domain and three characterized Small Ubiquitin-like Modifier (SUMO) interacting motifs (SIMs; Figure 3A) (17,41). SUMO, similarly to ubiquitin, is a small protein that is covalently attached to lysine residues within proteins, a post-translational modification (PTM) that can act to modify the localization and/or function of the target protein (42). SUMOylation is known to be involved in the DDR, including through the activities of the SUMO E3 ligases PIAS1 and PIAS4 that localize to DNA breaks and promote DSB repair (42–46). MYM-type ZnFs, including those found within ZMYM2, have also been demonstrated to bind to SUMO (31). Given the multiple domains within ZMYM2 that could mediate interactions involved in DNA damage recognition and recruitment, we performed deletion mapping of GFP-tagged ZMYM2 to identify regions of ZMYM2 required for its localization to DNA damage sites using laser micro-irradiation and live-cell fluorescence confocal microscopy.

We previously identified the TRASH domain of ZMYM3 as a region that binds double-stranded DNA and promotes DNA damage localization of this protein (17). A related domain is present in ZMYM2 based on the Protein Homology/analogY Recognition Engine - Phyre2 (http://www.sbg.bio.ic.ac.uk/phyre2/). This putative DNA-binding TRASH domain of ZMYM2 is highly conserved across various species (Supplementary Figure S4A). Interestingly, deletion of this TRASH domain within ZMYM2 had no measurable effect on the translocation of ZMYM2 to damage sites (Figure 3B and Supplementary Figure S4A). The expression of the ZMYM2 ΔTRASH was similar to WT ZMYM2, ruling out the potential of protein stability to impact these results (Figure 3C). This observation was unexpected given that ZMYM3 requires the TRASH domain for recruitment to damage sites (17). In order to explore this question further, we compared the amino acid sequence of the canonical TRASH domain with those found in ZMYM2 and ZMYM3. Since our previous analysis showed that the TRASH domain of ZMYM3 associated with DNA without the C-terminal MYM motif that is included in the TRASH domain region (17), we removed the MYM motif from the TRASH domain for our analysis. Interestingly, sequence comparisons of TRASH domains revealed several residues that were not conserved between ZMYM2 and ZMYM3 (Supplementary Figure S4B). In addition, using the DRNApred computational DNA/RNA binding prediction tool (47), the residues within the TRASH domain of ZMYM2 displayed a low DNA-binding probability (Supplementary Figure S4B). Conversely, the TRASH domain of ZMYM3 had a high DNA-binding probability, which is consistent with our demonstration that this domain within ZMYM3 binds double-stranded DNA (17). Taken together, our analysis suggests that the TRASH domain of ZMYM2 is unlikely to bind DNA, an idea further supported by the dispensable nature of this domain for DNA damage recruitment of ZMYM2 (Figure 3B).

Unlike the TRASH domain of ZMYM2, deletion of the ZnF domains within ZMYM2 resulted in reduced localization of this mutant ZMYM2 protein to damage sites compared to WT ZMYM2 (Figure 3D; protein expression of ZMYM2 ΔZnF shown in C). Based on the potential involvement of SUMO in regulating the DNA damage functions of ZMYM2, we set out to further investigate the importance of SUMO binding by ZMYM2 by mutating each of the three individual SIM domains within ZMYM2, which we accomplished by replacing four residues of the core hydrophobic motifs known to be required for SUMO binding with alanine residues in each SIM (Figure 3A) (41). Surprisingly, mutations within the defined SIM domains of ZMYM2 resulted in little to no effect on the accrual of ZMYM2 at DNA damage sites, which exhibited comparable localization kinetics to DNA breaks as WT ZMYM2 (Figure 3E; protein expression of ZMYM SIM mutants compared to WT shown in F). These results suggested the potential involvement of SUMOylation in the regulation of ZMYM2 damage recruitment through its ZnF but not SIM motifs. To further explore the involvement of SUMO in regulation ZMYM2 damage recruitment, we depleted PIAS1 or PIAS4, two key SUMO E3 ligases involved in the DDR (44,48), and tested ZMYM2 DNA damage localization in cells deficient for these SUMO ligases. While PIAS1-depleted cells supported ZMYM2 damage localization, which was indistinguishable compared to WT cells, PIAS4-deficiency almost completely abolished ZMYM2 recruitment to DNA damage sites (Figure 3G; efficiency of siRNA protein depletions is provided in Supplementary Figure S4C). Cells deficient for PIAS1 or PIAS4 also reduced ZMYM3 recruitment to damage sites (Supplementary Figure S4D). We also tested the involvement of the γH2AX effector MDC1, given its regulation by SUMO and involvement in promoting SUMOylation at DNA breaks upstream of PIAS1 and PIAS4 (44,45). Depletion of MDC1 reduced the engagement of ZMYM2 at DNA damage sites (Figure 3H). Altogether, these results revealed the involvement of SUMOylation by the PIAS1 and PIAS4 E3 SUMO ligases and MDC1 as well as potential SUMO binding by the MYM-type ZnFs within ZMYM2, in modulating ZMYM2 and ZMYM3 recruitment dynamics to DNA damage sites.

### ZMYM2 regulates BRCA1 and ABRA1 recruitment to DNA breaks

**Figure 4.**
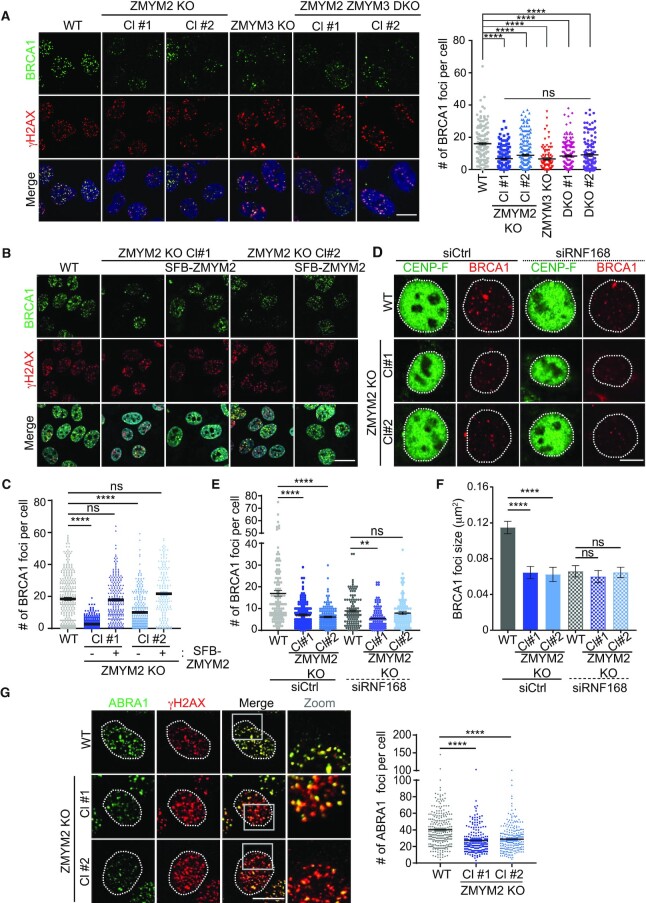
ZMYM2 is required for efficient BRCA1 engagement at DNA lesions. (**A**) BRCA1 foci formation following IR requires ZMYM2 and ZMYM3. WT, ZMYM2 KO, ZMYM3 KO and ZMYM2 ZMYM3 KO U2OS cells were treated with 10 Gy IR and analyzed by IF 3 h post-IR. γH2AX foci acts as positive control for IR-induced DSBs. Right panel: quantification of BRCA1 foci. Total number of BRCA1 foci per cell are indicated. All quantification data are represented as mean ± S.E.M. from > 100 from three independent experiments (*n* = 3), (****P < 0.0001, one-way ANOVA test). Scale bar = 10 μm. (**B**) Ectopic expression of ZMYM2 rescues BRCA1 foci formation in U2OS ZMYM2 KO cells. Experiments were performed in (A). (**C**) Quantification of (B). Data represented as mean ± S.E.M. from >90 cells, *n* = 2. Scale bar = 20μm. (**D**) IR-induced BRCA1 foci formed in S/G2 are reduced in ZMYM2 KO cells. Cells were treated with 10 Gy IR and BRCA1 foci were analyzed in CENP-F positive cells 6 h post-IR by IF. (**E**) Quantification of (D). All quantification data are represented as mean ± S.E.M. from >100 cells, *n* = 3. (**F**) Quantified BRCA1 foci size. BRCA1 foci sizes (area) were analyzed using ImageJ particle analysis. All quantification data are represented as mean ± S.E.M. from >100 foci, *n* = 3. (ns: not significant, **P < 0.01, ****P < 0.0001, one-way ANOVA test). Scale bar = 10 μm. (**G**) ABRA1 IR-induced foci are defective in ZMYM2 KO cells. Analysis and quantification (right panel) were performed as in (A). Data represented as mean ± S.E.M. from >200 cells, *n* = 2. Scale bar = 10 μm. (****P < 0.0001, one-way ANOVA test).

Given our identification of ZMYM2 as a DNA damage-localized protein involved in HR repair and cell survival following DNA damage including by IR, we next set out to further identify the molecular basis of these observations. We previously reported that the related protein, ZMYM3, functions to promote BRCA1 accrual at DSBs, an observation that helped to explain how loss of ZMYM3 negatively impacts HR repair (17). To test if ZMYM2 also regulates BRCA1 localization to DNA lesions, we measured BRCA1 foci formation in response to IR in WT and ZMYM2 KO cells. Compared to WT parental U2OS cells, BRCA1 ionizing radiation-induced foci (IRIF) formation was reduced in two independently generated ZMYM2 KO cell lines (Figure 4A). The extent of defective BRCA1 recruitment to IR-induced breaks was similar in ZMYM3 KO cells compared to ZMYM2 KO cells, suggesting that these ZnF-containing proteins may act in the same pathway to support BRCA1 accumulation at DNA breaks (Figure 4A). To further address this possibility, we generated ZMYM2 and ZMYM3 double mutant KO (DKO) cells (Supplementary Figure S5A). Consistent with ZMYM2 and ZMYM3 functioning in the same pathway in regard to BRCA1, analysis of BRCA1 IRIF formation in two independent ZMYM2 ZMYM3 DKO cell clones revealed an epistatic relationship of these genes in functioning to promote BRCA1 localization to DSBs. Indeed, the reduced ability of BRCA1 to form foci at IR-induced breaks observed in ZMYM2 KO, ZMYM3 KO or the double mutant KO cell lines was indistinguishable between these mutant cell lines (Figure 4A). We observed the same requirement for ZMYM2 in supporting BRCA1 localization to DSBs generated by the nuclease FokI in U2OS-DSB-reporter cells, which provided further evidence for the involvement of ZMYM2 in facilitating BRCA1 functions at break sites (Supplementary Figure S5B) (19). The involvement of ZMYM2 in facilitating BRCA1 break accrual was further validated in ZMYM2 KO cells by ectopically expressing ZMYM2, which was able to restore BRCA1 foci formation and complement the ZMYM2 gene-edited loss in this cell line to levels comparable to WT parental cells, thus ruling out any off-target effects that may be generated by CRISPR-Cas9 gene-editing (Figure 4B; quantified in C). Taken together, these results demonstrate that ZMYM2, together with ZMYM3, are required to support efficient engagement of BRCA1 with DSB sites.

BRCA1 associates with diverse DDR factors to form several complexes, which are involved in DSB repair via both DNA damage signaling and HR repair (49). BRCA1 recruitment to DSBs includes interactions that spread in large domains surrounding the break, which are dependent on the chromatin ubiquitination pathway which includes RNF168 (50,51). A recent study revealed that RNF168-depletion reduced the large DNA damage signaling associated BRCA1 IRIF whereby BRCA1 foci at the break were still observed, albeit smaller in size, in S/G2 phase cells (22). These smaller BRCA1 foci represent HR repair sites that are distinct from the larger BRCA1 foci that are dependent on the chromatin ubiquitination pathway. To determine which species of BRCA1 ZMYM2 supports at the damage site, we analyzed BRCA1 foci formation in WT and ZMYM2 KO cells in both RNF168 proficient and deficient conditions. Consistent with RNF168-mediated ubiquitination being required for 53BP1 retention at DSB sites (52), we observed that RNF168-depletion abolished 53BP1 IRIF formation in S/G2 cells in both WT and ZMYM2 KO cell (Supplementary Figure S5C and D). While BRCA1 IRIF were reduced in ZMYM2 KO cells compared to WT cells as expected, the small BRCA1 foci that remain in RNF168-depleted cells were similar in size in WT and ZMYM2 KO cells (Figure 4D–F). We observed a subtle reduction in BRCA1 foci number in ZMYM2 KO cells compared to WT cells upon RNF168 depletion although statistical significance was reached in only one of the two ZMYM2 KO clones analyzed (Figure 4D and E). These data suggest that ZMYM2 may play a more prominent role in regulating BRCA1 recruitment to the regions surrounding the damage site that are involved in DNA damage signaling rather than regulating BRCA1 resection and HR functions at the break site. These data are consistent with the fact that RNF168-deficient cells display defects in HR (22), suggesting that BRCA1 and potentially other DDR factors that load onto chromatin that surrounds the break also ultimately participate in promoting efficient repair of DSBs by HR.

**Figure 5.**
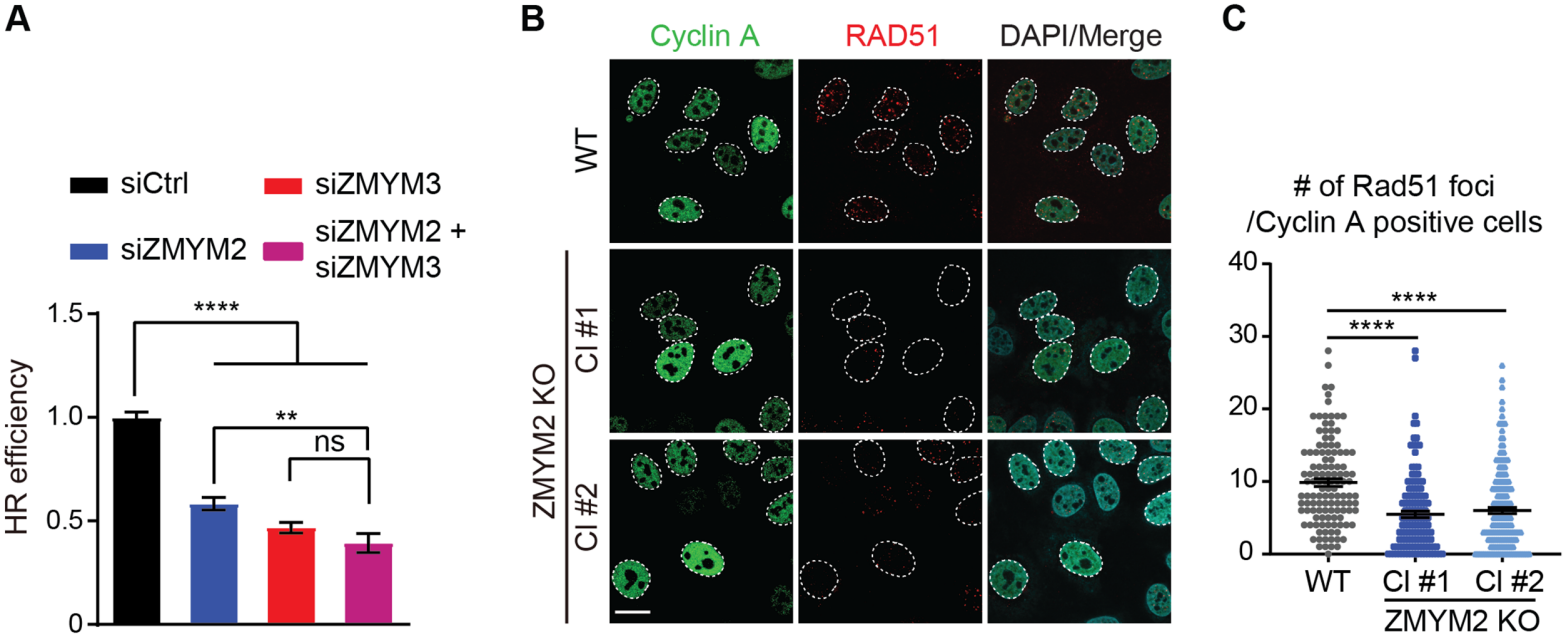
ZMYM2 functions in concert with ZMYM3 to promote HR repair. (**A**) Efficiency of HR repair by DR-GFP cell-based assay. U2OS DR-GFP cells were treated with either control or gene-specific siRNAs (ZMYM2 and/or ZMYM3). GFP + cells (HR-events) were identified by FACS analysis. HR efficiencies were normalized to siCtrl cells. Data are represented as mean ± S.E.M., *n* = 3, (ns; not significant, **P < 0.01, ****P < 0.0001 one-way ANOVA test). (**B, C**) IR-induced RAD51 foci are reduced in ZMYM2 KO U2OS cells. WT and ZMYM2 KO cells were treated with IR (10 Gy) and analyzed by IF 3 h post-treatment. Cells were stained with cyclin A to identify S and G2 cells. Quantification of RAD51 foci in cyclin A-positive cells in (**C**). Data represented as mean ± S.E.M. from >120 cells, *n* = 3 (****P < 0.0001, one-way ANOVA test). Scale bar = 20 μm.

In addition to regulating BRCA1 recruitment to DNA lesions, ZMYM3 interacts with RAP80 and ABRA1, two additional members of the BRCA1-A complex (17). RAP80 and ABRA1 also accumulate at DNA lesion and control the recruitment of BRCA1 to damage sites (53–56). To determine the relationship between ZMYM2 and these factors, we assessed their ability to form IRIF in WT and ZMYM2 KO cells. ABRA1 displayed strongly reduced recruitment to IR-induced DSBs in cells lacking ZMYM2 while RAP80 IRIF were unaffected by the loss of ZMYM2 (Figure 4G; Supplementary Figure S5E). These results are consistent with ZMYM2 and ZMYM3 functioning in the same pathway as ZMYM3 functions downstream of RAP80 at damage sites and is also required for ABRA1 recruitment to DSBs (17). A previous study showed that the depletion of MERIT40, another member of the BRCA1-A complex, reduced BRCA1-A complex formation by disrupting the stability of the BRCA1-A complex components (57). Analysis of both total protein levels of RAP80, BRCA1 and ABRA1 as well as complex formation as determined by RAP80 coimmunoprecipitation (co-IP) analysis of these factors revealed no appreciable differences in either protein levels or BRCA1-A complex stability in either ZMYM2 or ZMYM3 KO cells compared to WT cells (Supplementary Figure S5F). These results suggest that the reduced ABRA1 and BRCA1 accrual at DSBs in ZMYM2- or ZMYM3-deficient cells is not due to the reduced stability of the BRCA1-A complex upon ZMYM2 loss. Furthermore, co-depletion of ZMYM2 and ZMYM3 reduced HR similarly to either single protein depletion, suggesting that these factors function in the same pathway to orchestrate HR repair (Figure 5A). BRCA1 is required for loading the RAD51 recombinase onto resected DSBs to promote HR repair (58). Consistent with defects in BRCA1 damage recognition and HR repair in cells lacking ZMYM2, RAD51 IRIF formation in ZMYM2 KO cells were also reduced in cyclin A positive cells, a marker for the S/G2 cell cycle phases where HR preferentially occurs (Figure 5B and C). These data are in line with the differences observed in HR-associated BRCA1 foci in ZMYM2 KO cells compared to WT control cells, which may contribute to these downstream effects. Taken together, these data reveal that ZMYM2 in concert with ZMYM3 functions to promote BRCA1 and its associated factor ABRA1 accumulation at DSBs, as well as RAD51 loading, which helps explain the reduced HR repair efficiency observed in cells deficient for ZMYM2.

### ZMYM2 interacts with ZMYM3 to regulate its recruitment to DNA damage

**Figure 6.**
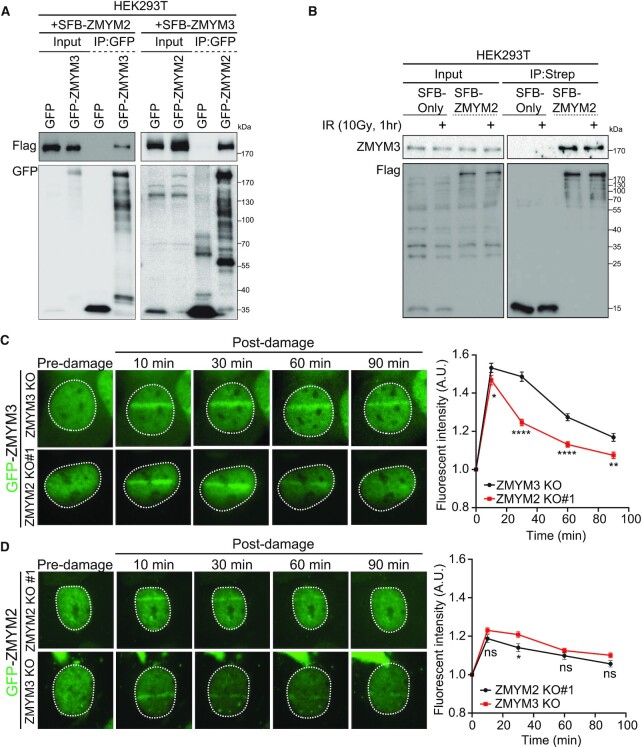
ZMYM2 interacts with ZMYM3 and regulates its association with DNA damage. (**A**) Co-Immunoprecipitation (co-IP) analysis identifies an interaction between ZMYM2 and ZMYM3. Differentially SFB and GFP-tagged ZMYM2 and ZMYM3 were co-expressed in HEK293T cells and IPed using anti-GFP beads followed by western blotting analysis with the indicated antibodies. GFP alone expressing cells were analyzed as controls. (**B**) Interactions between ZMYM2 and ZMYM3 are not altered by IR treatment. Samples from untreated or IR-treated HEK293T cells were obtained and analyzed by co-IP as in (A) with either SFB alone or SFB-ZMYM2 expressing cells. Endogenous ZMYM3 was detected in co-IP samples with a specific antibody. (**C, D**) ZMYM2 promotes retention of ZMYM3 at damage sites. U2OS ZMYM2 and ZMYM3 KO cells expressing GFP-tagged ZMYM3 or ZMYM3 were damaged using a UV-C laser and analyzed over time post-damage by live-cell fluorescence confocal microscopy. Right panel: quantification of GFP protein signal at the damage site over time (0–90 min. Data represent mean ± S.E.M., *n* ≥ 6 cells analyzed per condition (ns; not significant, *P < 0.05, **P < 0.01, ****P < 0.0001 by multiple unpaired *t*-test). Dotted white lines mark cell nuclei.

The phenotypic similarities between ZMYM2- and ZMYM3-deficient cells, as well as their shared membership in the ZMYM family of ZnF proteins, prompted us to further characterize their relationship to each other in the DDR. We previously identified ZMYM2 as an immunoprecipitated protein in ZMYM3 IPs by mass spectrometry, suggesting that ZMYM2 and ZMYM3 may interact with each other (17). To address this question further, we performed Co-IP western blotting analysis of both immuno-purified SFB-tagged ZMYM2 and ZMYM3 from cell lysates. This analysis confirmed the presence of ZMYM2 in ZMYM3 immuno-purified protein complexes and vice versa, as IP of ZMYM2 also co-purified ZMYM3 (Figure 6A). The relative concentration of ZMYM2 and ZMYM3 containing complexes was similar between untreated and IR-treated cells, suggesting that these interactions may occur constitutively, and not merely as a result of induced DNA damage (Figure 6B). We considered that interactions between ZMYM2 and ZMYM3 may govern their ability to recognize and be recruited to DNA lesions.

We therefore set out to directly address this possibility by testing the recruitment dynamics of both ZMYM2 and ZMYM3 to UV-A (355 nm) laser damage in BrdU-sensitized cells lacking ZMYM3 or ZMYM2 respectively. This analysis revealed that ZMYM2 and ZMYM3 were recruited independently from each other at early time points (i.e. <12 min post-laser damage) following laser damage (Supplementary Figure S6A and B). Interestingly, when we performed similar experiments using live-cell analysis for longer time intervals following DNA damage (i.e. 30, 60 and 90 min post-laser damage), we observed that the recruitment of ZMYM3 was reduced in ZMYM2 KO (Figure 6C). However, when we performed the reciprocal experiment with ZMYM2 recruitment in ZMYM3 KO cells, we did not detect any difference in recruitment dynamics between cells expressing or lacking ZMYM3 (Figure 6D). Similar results were obtained in fixed cells, which allowed DNA damage sites to be identified by γH2AX staining (Supplementary Figure S6C). Analysis of ZMYM3 recruitment to laser damaged revealed that 1 hr-post DNA damage, the ability of ZMYM3 to gather at damage sites was reduced in ZMYM2-deficient cells compared to ZMYM2-proficient WT cells. Collectively, these data revealed that ZMYM2 and ZMYM3 interact to form complexes containing both factors. Our results also show that ZMYM2 and ZMYM3 are recruited to DNA damage sites independently from each other but that the retention of ZMYM3 at DNA damage sites requires ZMYM2. These results also help clarify why cells deficient for ZMYM2 or ZMYM3 display similar defects in DNA repair processes.

### ZMYM2 antagonizes 53BP1 to facilitate HR repair

**Figure 7.**
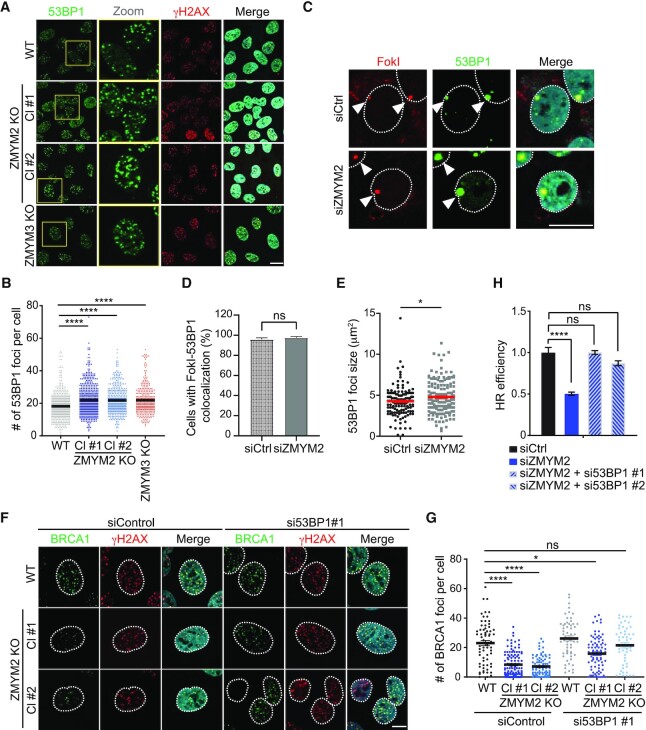
ZMYM2-deficiency increases 53BP1 engagement with DNA breaks. (**A**) Loss of ZMYM2 or ZMYM3 increases 53BP1 foci following IR-treatment. Foci formation of 53BP1 and γH2AX was analyzed following IR treatment (10 Gy, 3h) by IF in parental U2OS and ZMYM2 and ZMYM3 KO cells. (**B**) Quantification of (A). Data represented as mean ± S.E.M. from > 300 cells, *n* = 3, Scale bar = 20 μm, (****P < 0.0001, one-way ANOVA test). (**C**) 53BP1 is hyper-accumulated at FokI-mediated DSBs. U2OS mCherry-LacI-FokI DSB reporter cells transfected with indicated siRNAs were treated with 4-OHT and Shield-1 for >4 h to induce FokI-mediated DSBs. 53BP1 foci number and size were analyzed. (**D**) Quantification of (C). Data represent mean ± S.E.M., *n* = 3, (ns; not significant, two-tailed unpaired *t*-test). (**E**) 53BP1 foci sizes (area) were analyzed using ImageJ. Data represent mean ± S.E.M., from >120 cells, *n* = 3 (*P < 0.05 two-tailed unpaired *t*-test). (**F**) Defective BRCA1 foci formation in ZMYM2 KO U2OS cells is rescued by depletion of 53BP1. BRCA1 foci were analyzed by IF following IR-treatment (10 Gy. 3h) in WT and ZMYM2 KO cells (two independent KO clones; Cl#1 and Cl#2) treated with control siRNAs or siRNAs targeting 53BP1. (**G**) Quantification of (F). Data represented as mean ± S.E.M., *n* = 3, Scale bar = 10 μm (ns; not significant, ****P < 0.0001, one-way ANOVA test). (**H**) Depletion of 53BP1 rescues the HR deficiency in ZMYM2-depleted cells. HR levels were obtained in DR-GFP U2OS cells transfected with siRNAs targeting the indicated genes. Two-independent siRNAs targeting 53BP1 were analyzed. Data represent mean ± S.E.M., *n* = 3, (ns; not significant, ****P < 0.0001, one-way ANOVA test).

53BP1 has been reported to have an inhibitory effect on BRCA1 localization to damage sites, which has been proposed to occur through its ability to stabilize chromatin topologies around DNA breaks that limit DNA end-resection (9,59,60). Thus, we asked whether ZMYM2 deficiency affected 53BP1 localization to DNA breaks. Interestingly, we could observe an elevated association of 53BP1 at break sites following IR-treatment or FokI-generated DSBs in both ZMYM2 and ZMYM3 KO cells compared to WT cells (Figure 7A–D). We also observed an increase in the size of the 53BP1 focus associated with nuclease-generated breaks in ZMYM2-depleted cells compared to WT cells (Figure 7C and E). Increased 53BP1 foci frequency was also observed in ZMYM2 KO cells positive for G2 cell cycle phase marker CENP-F following IR damage (Supplementary Figure S5D). This is interesting as 53BP1 loading in S-phase is regulated by dilution of the H4K20me2 mark during DNA replication that allows binding of BRCA1–BARD1 to unmethylated H4 tails (61,62). Despite this additional chromatin regulatory pathway for repelling 53BP1 loading and increasing BRCA1 interactions with DSBs in S/G2 cells, increased 53BP1 retention at breaks sites was still observed in ZMYM2/3-deficient cells, including during G2. The detection of increased levels of 53BP1 at break sites is not explained either by altered protein levels as total 53BP1 protein levels were comparable between, ZMYM2 KO, ZMYM3 KO and parental WT cells (Supplementary Figure S7A). We were unable to observe a direct interaction between ZMYM2 or ZMYM3 with 53BP1, making it unlikely that 53BP1 recruitment is regulated by these ZnF proteins through direct binding with 53BP1 (Supplementary Figure S7B).

We next considered that the reduced levels of BRCA1 at DNA damage sites in ZMYM2 and ZMYM3 KO cells could be a result of 53BP1 hyper-accumulation at DNA breaks. We reasoned that depletion of 53BP1 may allow BRCA1 interactions with damaged DNA sites in ZMYM2 or ZMYM3-deficient cells. Indeed, depletion of 53BP1 using two different siRNAs in two independent ZMYM2 KO cell lines resulted in a significant rescue of BRCA1 foci formation in response to IR in these cells compared to ZMYM2 KO cells alone (Figure 7F, G and Supplementary Figure S7C, D). Moreover, depletion of 53BP1 in ZMYM2-depleted cells also restored HR repair efficiencies to levels near those obtained in WT cells transfected with non-targeting siRNAs as measured by the DR-GFP cell-based HR assay (Figure 7H). Thus, these results identified 53BP1 dysregulation at DNA breaks sites as the underlying cause leading to an inability of BRCA1 to localize to DNA breaks properly and defective HR repair in ZMYM2- and ZMYM3-deficient cells.

**Figure 8.**
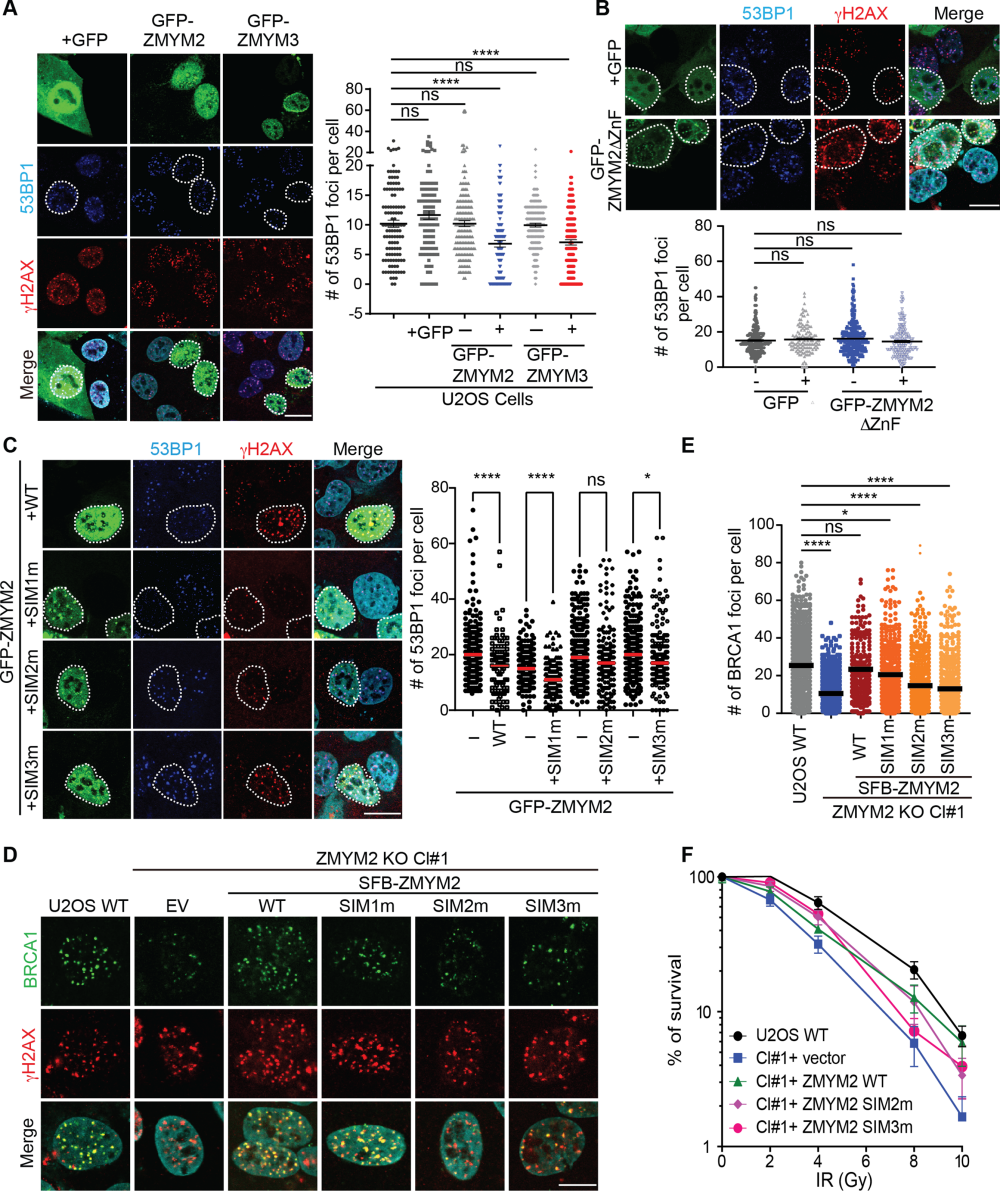
ZMYM2 antagonizes 53BP1 at damage sites via SUMO binding. (**A**) ZMYM2 and ZMYM3 overexpression reduces 53BP1 IRIF. U2OS cells expressing GFP, GFP-ZMYM2, or GFP-ZMYM3 were analyzed for 53BP1 and γH2AX foci formation following IR treatment (10 Gy, 3h) by IF. Right panel: quantification of total number of 53BP1 foci per cell after IR treatment. Data represented as mean ± S.E.M. from >90 cells, *n* = 3, scale bar = 20 μm, (ns; not significant, ****P < 0.0001, one-way ANOVA test). (**B**) The ZnF domains of ZMYM2 are required to antagonize 53BP1 at IR-induced breaks. Analysis was performed as in a with GFP or GFP-ZMYM2ΔZnF expressing U2OS cells. lower panel: quantification of total number of 53BP1 foci per cell after IR treatment. Data represented as mean ± S.E.M. from >120 cells, *n* = 3, Scale bar = 20 μm (ns; not significant, one-way ANOVA test). (**C**) ZMYM2 SIM2 and SIM3, but not SIM1, are required to inhibit 53BP1 IRIF. Analysis was performed as in (A). Data represented as mean ± S.E.M. from >100 cells, *n* = 3 (ns; not significant, *P < 0.05, ****P < 0.0001, one-way ANOVA test). (**D**, **E**) ZMYM2 SIM2 and SIM3 mutants, unlike WT ZMYM2, are unable to complement deficient break association of BRCA1 that is observed in ZMYM2 KO cells. Experiments were performed as in Figure 4B. Quantification of number of BRCA1 foci per cell in each sample is provided in (E) (ns; not significance, *P < 0.05, ****P < 0.0001, one-way ANOVA test). (**F**) IR sensitivity of ZMYM2 KO cells complemented with WT ZMYM2 and derivatives. Experiments were performed as in Figure 2C with the indicated ZMYM2 WT and mutant clones.

In view of these results, we considered that ZMYM2 and ZMYM3 may directly compete with 53BP1 at DNA break sites. To test this idea, we overexpressed GFP-tagged ZMYM2 or ZMYM3 and analyzed the ability of 53BP1 to accumulate at IR-induced DSBs. Overexpression of either GFP-ZMYM2 or GFP-ZMYM3 reduced 53BP1 foci formation by ∼2-fold compared to untransfected cells or GFP only overexpressing cells (Figure 8A). These results were consistent with the notion that ZMYM2 and ZMYM3 compete with 53BP1 at damage sites, which helps to explain why BRCA1 recruitment is defective in cells deficient for either of these two ZnF proteins. We next considered which domains within ZMYM2 are required for antagonizing 53BP1 at DNA break sites. Using our competition assay for 53BP1 IRIF, we expressed several different ZMYM2 derivatives to identify the mechanistic basis for its ability to inhibit 53BP1 at IR-induced DSBs. Interestingly, overexpression of ZMYM2 lacking the ZnF domains was unable to exclude 53BP1 from break sites (Figure 8B). This result suggested that the inhibitory effect of ZMYM2 on 53BP1 break recruitment occurs at DNA breaks since ZMYM2 lacking its ZnF domains is unable to be recruited to DNA break sites. An alternative explanation could be that the ZnF domain mediates a binding event that is required for competing with 53BP1.

Given that the MYM-type ZnF domains within ZMYM2 are known to bind SUMO (31,41), we considered that SUMO-binding may be involved in this process. Our previous analysis of individual SIM domain mutations within ZMYM2 revealed that SIM1-3 are largely dispensable for damage recruitment of ZMYM2. However, expression of ZMYM2 containing either SIM2 or SIM3 mutations were unable to antagonize 53BP1 at break sites in response to IR while WT or SIM1 mutant ZMYM2 reduced 53BP1 IRIF formation (Figure 8C). Consistent with these results, WT and SIM1 mutant ZMYM2 were able to complement ZMYM2 KO cells and restore BRCA1 localization to DNA breaks with ZMYM2 SIM2 and SIM3 mutants failed to rescue this defect (Figure 8D and E). Colony survival assays following IR of these complemented cells provided consistent results, with expression of WT ZMYM2 in ZMYM2 KO cells rescuing IR sensitivity unlike vector only or either SIM2 and SIM3 mutant ZMYM2, which failed to support cellular survival to IR treatment to the same level as WT complemented ZMYM2 KO cells (Figure 8F). Taken together, these results revealed that ZMYM2 and ZMYM3 inhibit 53BP1 engagement with DNA break sites to allow BRCA1 recruitment and HR repair, including through the ability of ZMYM2 to interact with SUMO at DNA break sites. However, a reciprocal inhibition of ZMYM2 or ZMYM3 by 53BP1 was not observed as recruitment of ZMYM2 and ZMYM3 to DNA damage sites was similar in WT and 53BP1 KO cells (Supplementary Figure S7E; quantified in F). Mechanistically, our results revealed SUMO-binding by ZMYM2, both at the level of DNA damage recruitment and 53BP1 inhibition, as key interactions that allow ZMYM2 to restrict 53BP1 engagement at break sites, which is required to support efficient BRCA1 recruitment and subsequent HR repair of DSBs.

## DISCUSSION

**Figure 9.**
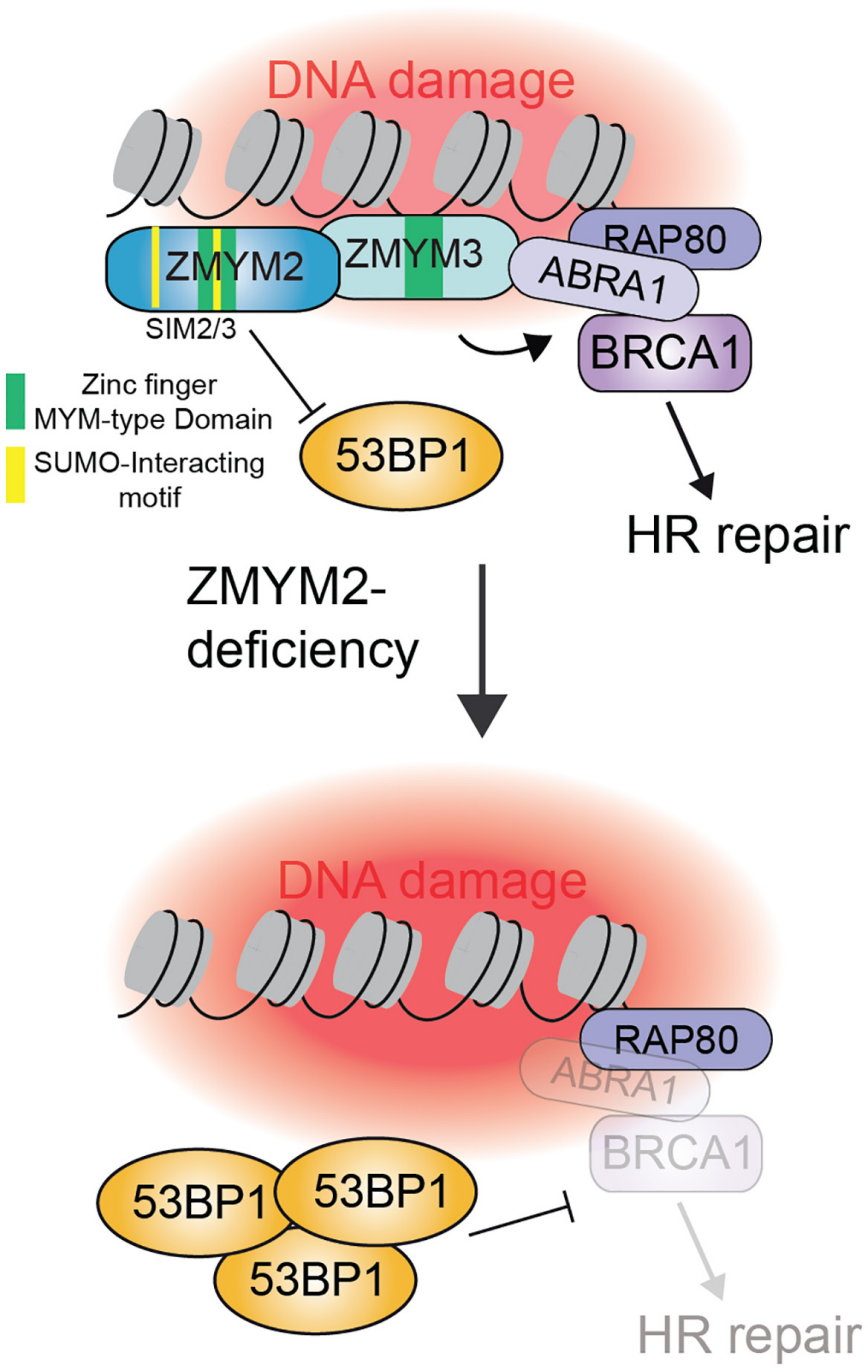
Model for ZMYM2 functions in promoting HR repair of DSBs. The ZnF proteins ZMYM2 and ZMYM3 localize to damaged DNA lesions where they function to antagonize 53BP1. ZMYM2 is an effector of SUMOylation at break sites, where it localizes to DSBs in a PIAS4-dependent manner that also involves its ZnF domains. Once localized to damage sites, the SIM2 and SIM3 domains of ZMYM2 are required to inhibit break-associated 53BP1 in a SUMO-binding dependent mechanism. In cells lacking this pathway, including ZMYM2-deficient cells, 53BP1 is hyper-loaded onto break sites where it out competes BRCA1, resulting in defective HR repair. This work identifies a new pathway critical for balancing the chromatin association of BRCA1 and 53BP1 to promote efficient DSB repair in human cells.

The human MYM-family of ZnF proteins contains 6 members and their functions are poorly understood. Here, by surveying the ability of all family members to be localized to DNA damage sites and support DSB repair, we have identified ZMYM2 as a new DNA damage response factor involved in the regulation of homologous recombination. Previous work had hinted at the potential involvement of ZMYM2 in the repair of DNA lesions generated by UV as cells expressing a ZMYM2-FGFR1 fusion protein exhibited UV sensitivity (34). However, our analysis did not reveal any overt defects in repair synthesis or cell sensitivity following UV damage in cells lacking ZMYM2 (Supplementary Figure S3). Rather, ZMYM2-FGFR1 may affect UV responses specifically as a fusion protein or in a specific genetic background. Our work has instead identified a role for ZMYM2 in regulating DSB repair through both NHEJ and an ability to constrain the NHEJ facilitator protein 53BP1 to favor the accumulation of BRCA1 onto chromatin, which is required to support HR repair. Our findings also lead to a model indicating the involvement of SUMOylation, including SUMO-binding by ZMYM2, in allowing the recruitment of ZMYM2 to DNA breaks and its ability to antagonize 53BP1 to allow HR repair of DSBs (Figure 9).

SUMOylation is a key PTM involved in regulating DNA double-strand break repair (42). Many key DDR factors involved in both HR and NHEJ have been identified as SUMOylated proteins, although how these modifications function to regulate DSB repair remains an active area of investigation (42,63,64). Our identification of ZMYM2 and SUMOylation in regulating BRCA1 recruitment to damage sites by their ability to antagonize 53BP1 provides additional insights into how SUMO functions in DSB repair. ZMYM2 contains two unique SUMO-binding motifs, including three SIM domains and nine MYM-type ZnFs, which both bind SUMO (41,65). Mechanistically, our findings revealed that SUMOylation functions in at least two distinct steps to regulate ZMYM2 DDR activities. ZMYM2 recruitment to DNA damage sites requires the SUMO E3 ligase PIAS4 as well as its ZnFs. Interestingly, engagement of ZMYM2 with DNA damage was not dependent on any of its 3 known SIM domains. Rather, ZMYM2 SIM2 or SIM3 mutants were unable to antagonize 53BP1 recruitment at damage sites. Thus, in addition to promoting recruitment of ZMYM2 to DNA lesions in a manner independent of SIM domains, these results suggest an additional role for SUMOylation downstream of damage localization in regulating ZMYM2 binding of SUMOylated proteins though the SIM2 and SIM3 domains that mediate 53BP1 inhibition at DNA lesions that is required to allow BRCA1 DSB recruitment and HR repair.

Hundreds of proteins are known to be SUMOylated and/or contain SIMs, making target identified in SUMO-dependent pathways challenging. It is worth noting that while depletion of PIAS4 reduced 53BP1 foci formation at damage sites (44), loss of ZMYM2 results in increased 53BP1 interactions at DNA lesions (Figure 7A–E). These results highlight the complex nature of SUMO signaling at break sites, which is required to both recruit and constrain 53BP1 interactions with damage sites, a function for SUMO and ZMYM2/3 identified here (Figure 8A–C). Based on our data, ZMYM2 is unlikely to regulate 53BP1 recruitment to damage sites through direct binding to SUMOylated 53BP1. Indeed, we have been unable to detect a direct interaction between ZMYM2 and 53BP1 even though 53BP1 has been reported to be SUMOylated by PIAS4 (Supplemental Figure S7B and (44)). Additional SUMOylated factors that regulate 53BP1 and BRCA1 DNA damage signaling are also known, including H2AX, MDC1, RNF8, RNF168, RAP80, as well as BRCA1 itself. Indeed, H2AX has multiple lysine residues that are SUMOylated by PIAS4 although to date, no known interactors have been identified (66). Interestingly, ZMYM3 interacts with BRCA1-associated factors including ABRA1 and its recruitment to DNA damage sites is reliant on both RAP80 and H2AX (17). Of interest, MDC1 is SUMOylated on lysine 1840 by PIAS4 (67). A non-SUMOylated MDC1 mutant results in increased MDC1 at break sites and reduced RAD51 foci formation and HR repair. Importantly, the defects in RAD51 loading at breaks and HR repair can be rescued by depletion of 53BP1. These results are very reminiscent of the defects observed in ZMYM2 and ZMYM3 deficient cells where BRCA1 foci formation and HR repair are defective and can be rescued by removing 53BP1 (Figure 7). It is tempting to speculate then that ZMYM2 and ZMYM3 SUMO-mediated functions involved in antagonizing 53BP1 may function through SUMO modified MDC1. While we observed a requirement for MDC1 to promote ZMYM2 recruitment to DNA damage sites (Figure 3H), future studies are warranted to investigate the molecular underpinnings of these observations and test the involvement of SUMOylation in regulating DSB repair pathway choice by 53BP1 and BRCA1, including through the involvement of ZMYM2 and ZMYM3.

Spatiotemporal dynamics of DDR factors are crucial to orchestrate the DDR factor dynamics and functions at DNA breaks (68–70). In addition to the fine-tuning of 53BP1 and BRCA1 interactions with DNA damage that is governed by ZMYM2 and ZMYM3, several other regulatory mechanisms of 53BP1 accrual at chromatin in the absence or presence of DNA damage have been reported. L3MBTL1 and JMJD2A interact with H4K20me2 in undamaged chromatin and prevent 53BP1 from binding to the histone mark (71,72). Nuclear mitotic apparatus protein (NuMA), Tudor interacting repair regulator (TIRR), forkhead box K1 (FOXK1), and AHNAK directly bind to 53BP1 and negatively regulate its interactions with damage sites while another 53BP1 associated molecule, LC8/DYNLL1, promotes 53BP1 foci formation (73–77). Moreover, other factors including RNF169, poly(ADP-ribose) polymerase 2 (PARP2), G9a-like protein (GLP) and Speckle-type POZ protein (SPOP) influence 53BP1 deposition at DNA lesions (78–81). Given the multi-valent interactions that orchestrate 53BP1 interactions with damage sites including binding to γH2AX, H2AK15ub, H4K20 methylation and the nucleosome acidic patch, there are several pathways whereby 53BP1 accrual at sites of DNA lesions can be regulated. This is also the case for BRCA1, which along with its binding partner BARD1, also exhibit several binding sites on damaged chromatin including unmethylated H4K20, H2AK15ub, nucleosome acidic patch and nucleic acid binding (reviewed in 82). Given the ability of ZMYM3 to interact with histones and bind DNA (17), we cannot rule out that the regulation of DSB repair by these ZnF proteins also occurs through chromatin interactions in addition to SUMO binding. Given the complexity of the interactions that orchestrate 53BP1 and BRCA1 function on chromatin at damage sites, future investigations of additional factors that regulate these DSB proteins on chromatin may shed light on additional mechanisms by which ZMYM proteins promote genome integrity.

The ability of ZMYM2 and ZMYM3 to support DSB repair and genome integrity may have implications in human diseases where ZMYM2 and ZMYM3 mutations have been identified. Indeed, both ZMYM2 and ZMYM3 have been implicated in several human diseases including cancer. Recurrent loss of function mutations of ZMYM3 have been identified in several cancers including chronic lymphocytic leukemia, medulloblastoma, Ewing sarcoma and pediatric cancers (83–88). In support of cancer mutations in ZMYM3 impacting the DDR, we previously showed that a R1274Q mutation identified in a tumor abolished the interaction between ZMYM3 and RAP80 (17). ZMYM2 has also been implicated in cancer as a fusion protein with the Fibroblast growth factor receptor 1 (FGFR1) in 8p11 myeloproliferative syndrome (EMS), where it constitutes ∼ 50% of all cases of FGFR1-fusion related myeloid/lymphoid neoplasms (89–91). In this disease, ZMYM is fused at the C-terminus with the FGFR1 resulting in a constitutively active and cytoplasmic fusion, abolishing the predominately nuclear localization of ZMYM2. Mutations in ZMYM2 have recently been reported in congenital anomalies of the kidney and urinary tract (CAKUT) (92). Fourteen out of nineteen CAKUT patients were found to contain a loss of function ZMYM2 mutation that failed to localize ZMYM2 to the nucleus (92). ZMYM2 mutations have also been observed in other cancers including uterine corpus endometrial carcinoma (UCEC) and non-small cell lung cancer (NSCLC) (93,94), including as a cancer driver gene in UCEC (93). We found that ZMYM2 mutations identified in UCEC are nonsense mutation and/or non-conservative missense mutations (TCGA). Considering our identification of ZMYM2 as a DDR factor involved in maintaining genome integrity, we speculate that ZMYM2 mutations may result in genome instability, a known contributing factor involved in cancer. In addition, targeting DDR pathway deficiencies is a promising strategy for cancer treatment interventions, including both the use of DNA damaging agents as treatments and/or using targeted therapies in HR-deficient tumors with PARP inhibitors (95–97). Our findings reported here provide the rationale for investigating HR-deficiency in ZMYM2 and ZMYM3 mutant tumors, which may provide new therapeutic strategies to target these pathways when dysregulated.

## DATA AVAILABILITY

The data generated in this study are available from the corresponding authors upon request.

## Supplementary Material

gkac160_Supplemental_File
