# Supplementary material for: ZMYM2 restricts 53BP1 at DNA double-strand breaks to favor BRCA1 loading and homologous recombination
Source: Nucleic Acids Res. 2022 Mar 7;50(7):3922–43. doi: 10.1093/nar/gkac160 (PMC9023290; doi:10.1093/nar/gkac160)
Supplement: gkac160_Supplemental_File [file gkac160_supplemental_file.pdf]

Supplementary Information

ZMYM2 restricts 53BP1 at DNA double-strand breaks to favor BRCA1 loading and homologous recombination

Doohyung Lee[1], Katja Apelt[2], Seong-Ok Lee[3], Hsin-Ru Chan[1], Martijn S. Luijsterburg[2], Justin W.C. Leung[3,*] & Kyle M Miller[1,4*]

Supplementary Figures S1-S7 with legends

Supplementary Figures

Supplementary Figure S1

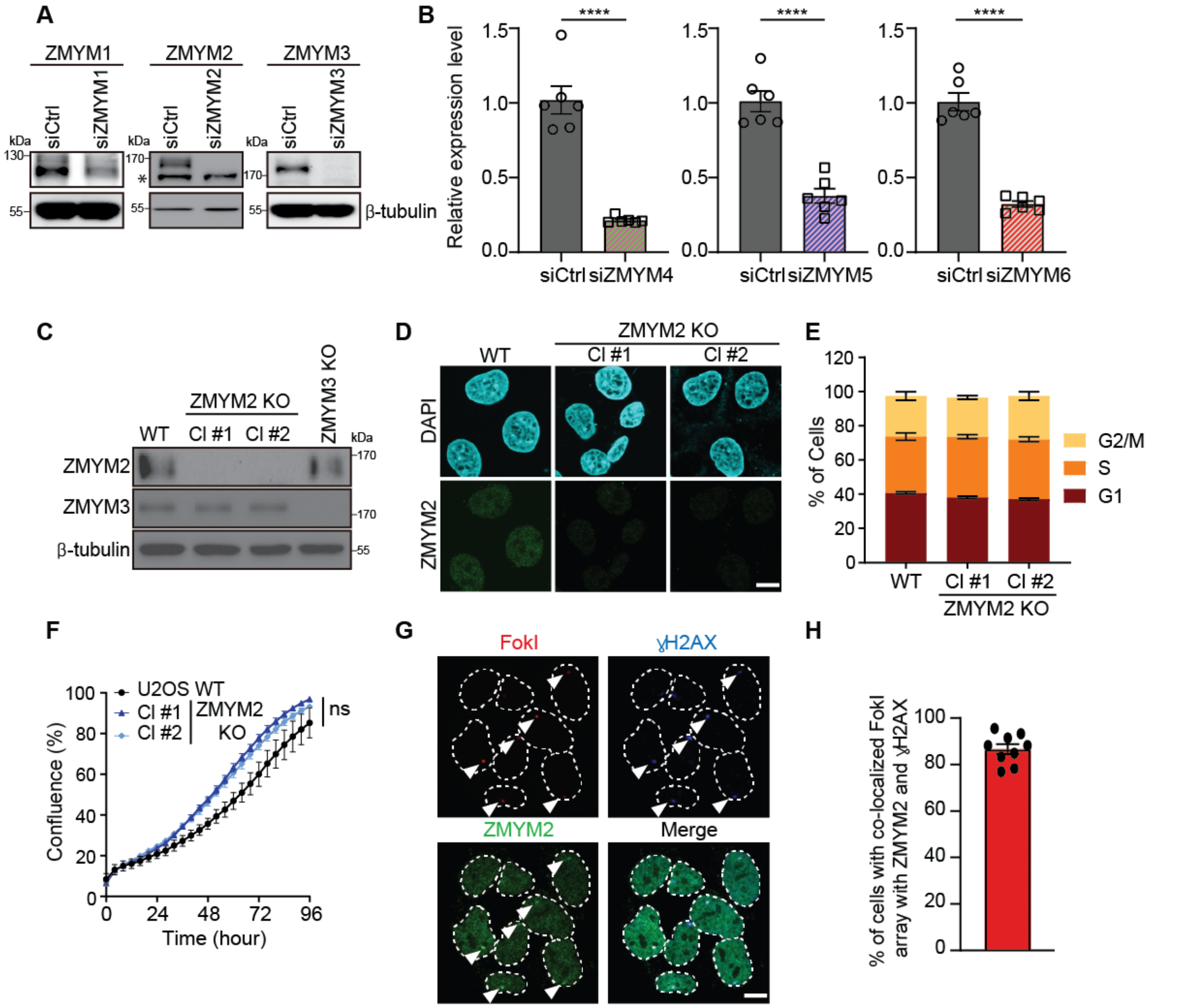

Supplementary Figure 1. Characterization of ZMYM2-deficient U2OS cells. **A, B** Validation of siRNA-mediated depletion of ZMYM proteins. U2OS cells were treated with siCtrl or siRNAs targeting individual ZMYM genes followed by analysis of knockdown efficiency by western blotting in **A** or RT-qPCR in **B**. In **B**, error bars represent mean ± SEM and n = 6; (****p<0.0001, two-tailed unpaired t-test). **C** ZMYM2

KO cell lines and endogenous ZMYM2 antibody validation by western blotting in **C** and immunofluorescence in **D**. **E** Cell cycle profile analysis of U2OS WT and ZMYM2 KO cells by flow cytometry. **F** Cell proliferation rates of U2OS WT and ZMYM2 KO cells are similar. Analysis was performed using an Incucyte. (n = 3, ns; not significant, One-way ANOVA test). **G** Endogenous ZMYM2 co-localized with the DNA damage marker γH2AX at Fok**I**-generated DNA double-strand breaks (DSBs). Fok**I**-mediated DNA breaks were induced by the treatment of 4-OHT and Shield-1 in U2OS-DSB-reporter cells and localized using mCherry-Fok**I**. White arrows indicate Fok**I** foci and sites of DSB generation. **H** Quantification of **G,** Data represented as mean ± S.E.M., from >200 Fok**I**-expressing cells, n = 3. Scale bar, 10μm.

Supplementary Figure S2

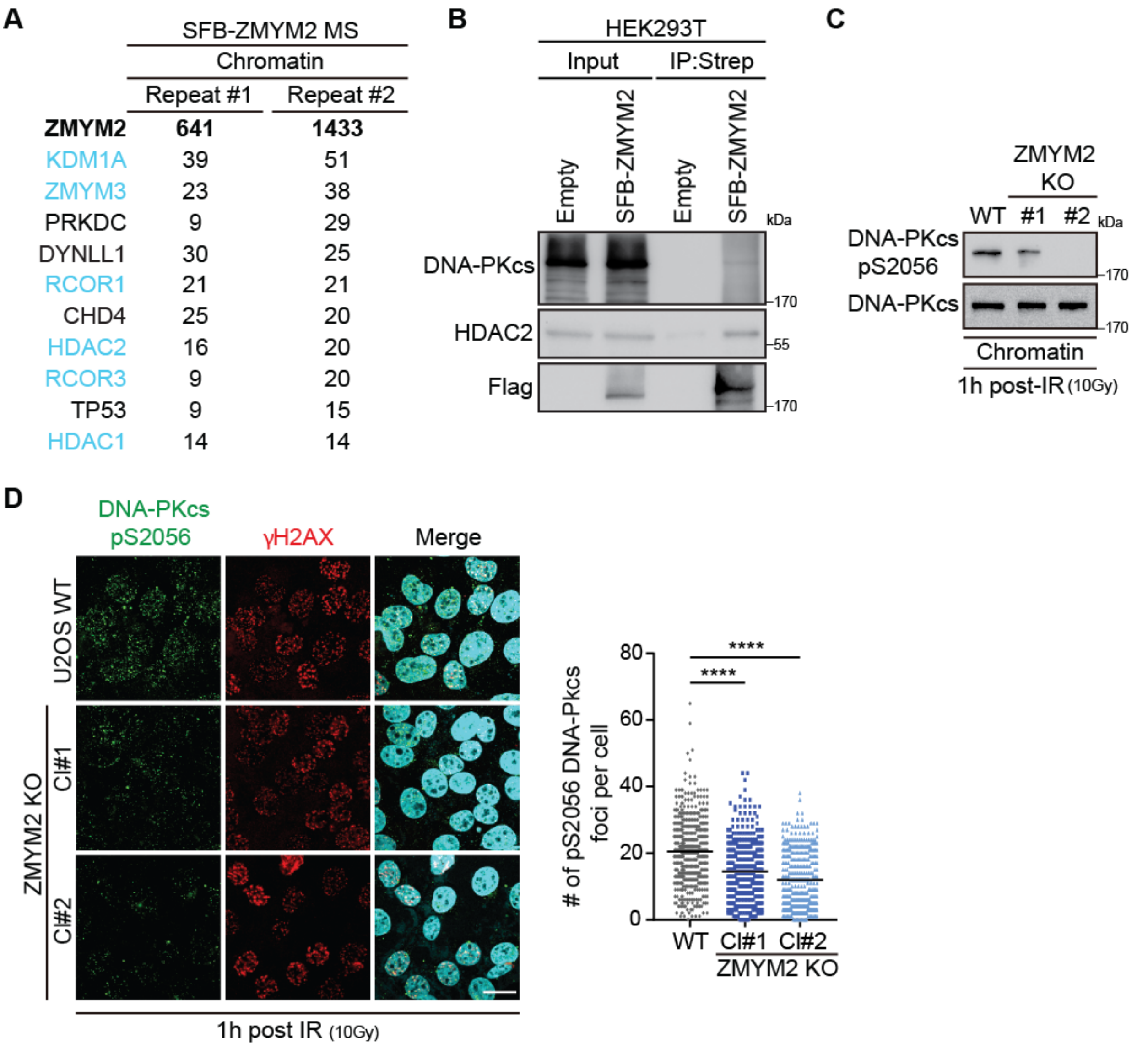

Supplementary Figure 2. Analysis of ZMYM2 in NHEJ. **A** Mass Spectrometry (MS) analysis of immune-purified ZMYM2 protein complexes. SFB-tagged ZMYM2 within the chromatin fraction was purified from HEK293T cells, and the co-purified complexes were analyzed by MS. ZMYM2 interacting proteins identified in 2 independent replicates containing at least 9 unique peptides are listed. Molecules highlighted with sky blue are known ZMYM2 interacting proteins. **B** ZMYM2 interacts with DNA-PK. HEK293T cells

were transiently transfected with empty vector or SFB-ZMYM2 vector. Proteins were purified using streptavidin and analyzed by western blotting with the indicated antibodies. **C, D** DNA-PK activation is reduced in ZMYM2 KO cells. U2OS WT and ZMYM2 KO cells were treated with IR (10 Gy) and samples analyzed 1 h post-IR treatment. DNA-PK phosphorylation levels were detected by western blotting in **C** and DNA-PK IRIF were analyzed using immunofluorescence in **D.** Data represented as mean ± S.E.M., from >150 cells, n = 3. Scale bar, 20μm.

Supplementary Figure S3

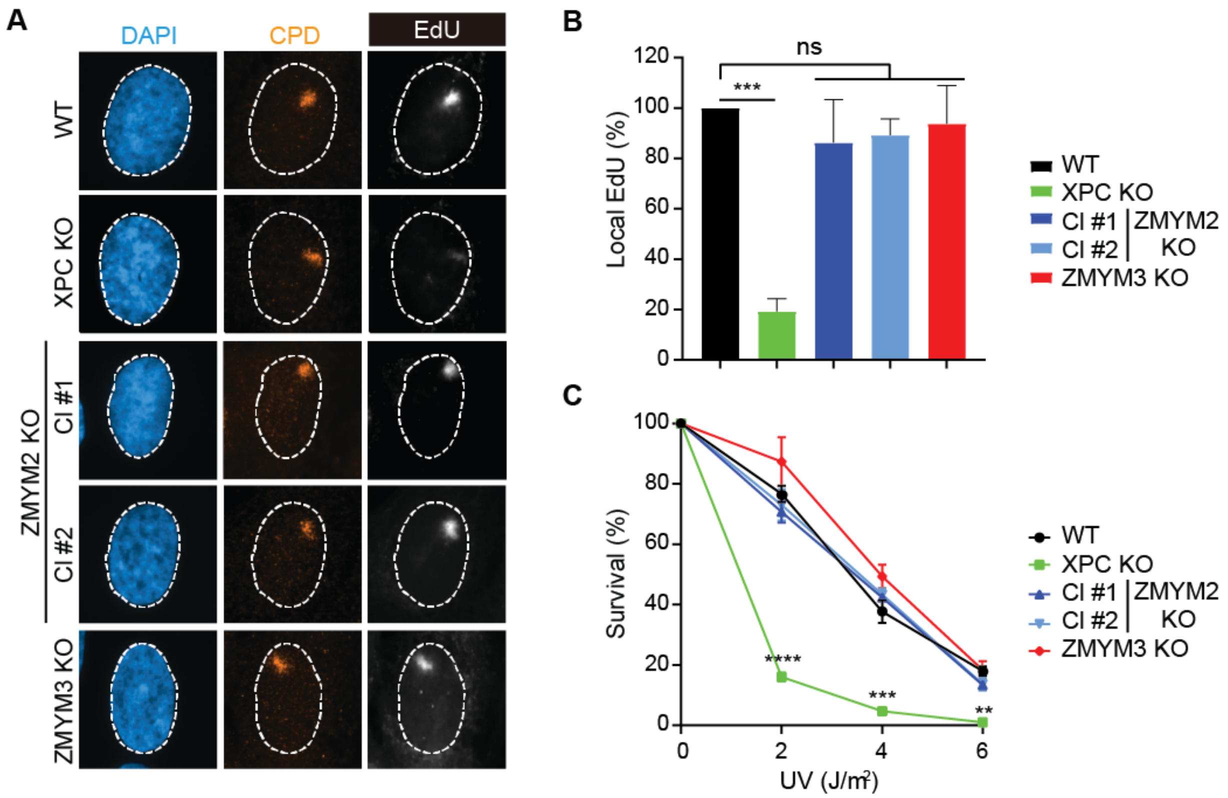

Supplementary Figure 3. ZMYM2 or ZMYM3 loss does not affect DNA synthesis inhibition after UV irradiation (30 J/m2) in U2OS cells. **A** Newly synthesized DNA was detected by EdU incorporation and U2OS XPC KO cells are a positive control for defective DNA synthesis regulation following UV damage. **B** Quantification of **A**. **C** ZMYM2 or ZMYM3 KO cells are not UV sensitive. U2OS cells with the indicated genotypes were treated with varying UV doses and cell survival was analyzed by colony formation assay. Data represented as mean ± S.E.M., n = 3. (ns; not significant, ***p<0.001. One-way ANOVA test).

Supplementary Figure S4

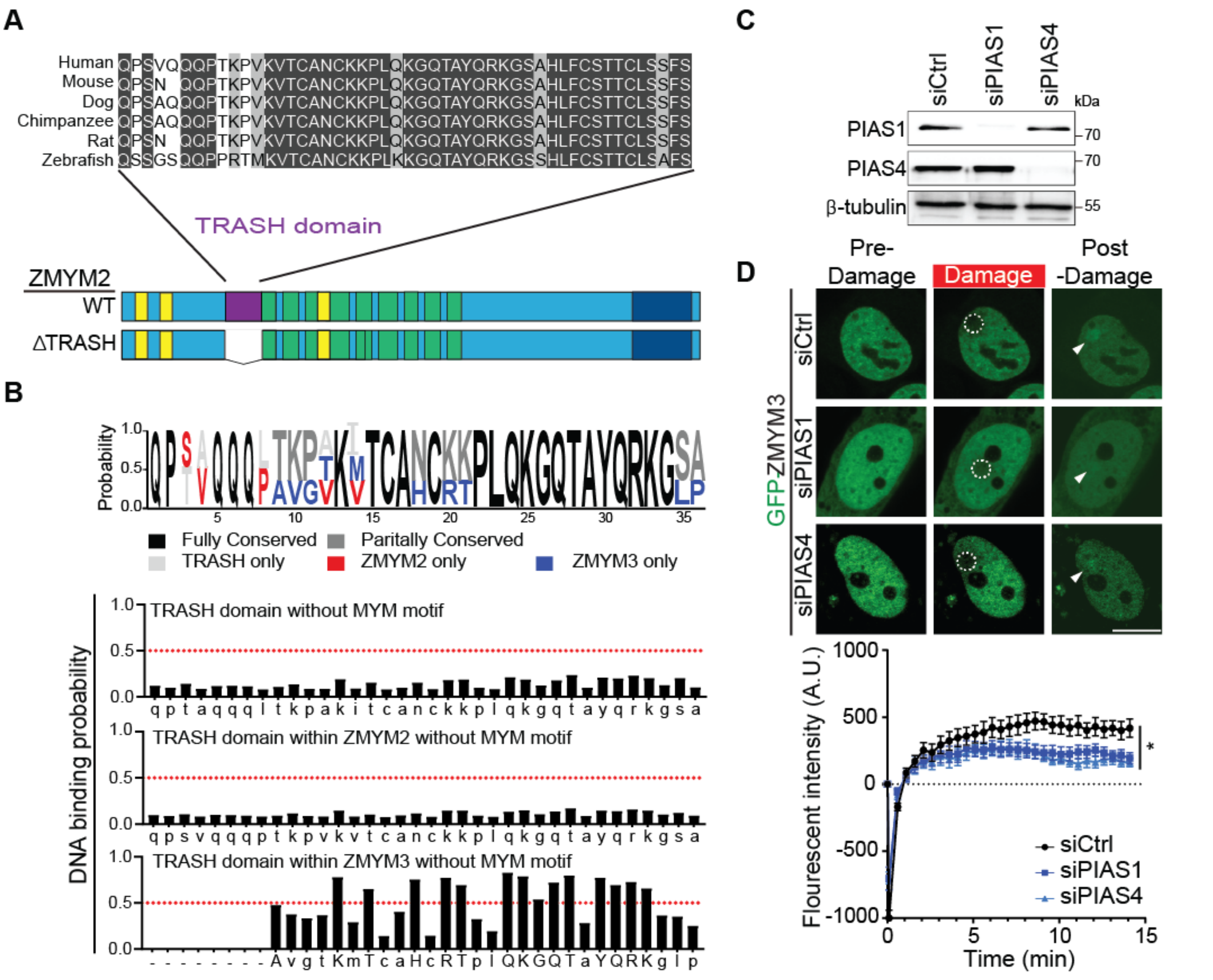

Supplementary Figure 4. Domain analysis of ZMYM2/3 and SUMO-dependent damage recruitment of ZMYM3. **A** ZMYM2 TRASH domain. ZMYM2 TRASH domain is highly conserved across several species. Schematic diagram of ZMYM2 domain structures (yellow - SIM, purple – TRASH, green – ZnFs, dark blue – DUF). **B** ZMYM2 TRASH domain exhibits low DNA binding probability compared to ZMYM3 TRASH domain. Comparison of amino acid sequences of the TRASH domain between the canonical TRASH domain and this region in ZMYM2 and ZMYM3. Lower panel: DNA

binding probability of TRASH domain within ZMYM2 and 3 without the MYM-type ZnF motifs. See Methods for details. **C** Validation of protein depletion by siRNA of PIAS1 and 4 by western blotting. U2OS cells were treated with siCtrl, siPIAS1, or siPIAS4 and analyzed by western blotting using the indicated antibodies. **D** PIAS1 and PIAS4 promote recruitment of GFP-tagged ZMYM3 to laser damage. Cells treated with indicated siRNAs were transfected with GFP-ZMYM3 and laser-mediated damage recruitment was analyzed 0-15 min post-damage by live-cell fluorescence confocal microscopy (quantified in bottom panel). Dotted white circles indicate damaged region and white arrowheads point to post-damaged area. Data represent mean ± S.E.M., n > 7 cells per condition (*$p<0.05$, two-tailed unpaired t-test). Scale bar, 10μm.

Supplementary Figure S5

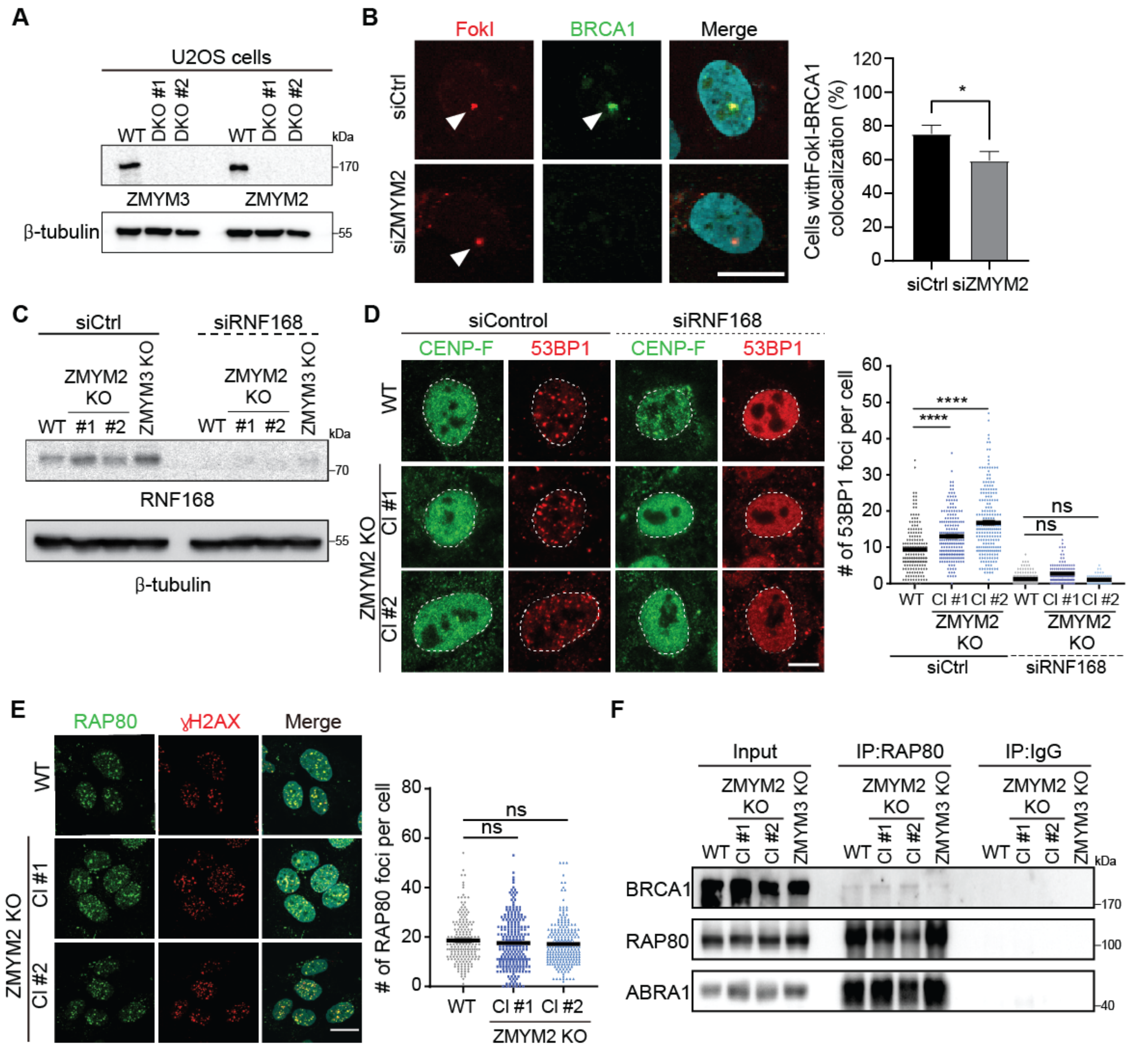

Supplementary Figure 5. Validation and analysis of ZMYM2 and ZMYM3 KO cells in the DNA damage response. **A** Validation of ZMYM2 ZMYM3 double knockout U2OS cells by western blotting. **B** ZMYM2 promotes recruitment of BRCA1 to FokI-mediated DSBs. Experiments were performed as in Supplementary Figure S1G. Cells transfected with the indicated siRNAs were immunostained with BRCA1 antibody, breaks detected

with mCherry-FokI and nuclear DNA detected with DAPI. Right panel: Quantification of BRCA1 colocalized with mCherry-FokI. Data represented as mean ± S.E.M. > 100 cells from n = 3. (*p<0.05, two-tailed unpaired t-test). White arrows indicate FokI foci and sites of DSB generation. Scale bar=20μm. **C** Validation of siRNA-mediated knockdown of RNF168 by western blotting. **D** 53BP1 IRIF are highly dependent on RNF168. Experiments were performed in WT and ZMYM2 KO cells as in **Figure 4D**. Right panel: quantification of 53BP1 foci in CENP-F positive cells after IR-treatment. Data represented as mean ± S.E.M. from > 100 cells, n = 3. (ns; not significant, ****p<0.0001, One-way ANOVA test). Scale bar=10μm. **E** RAP80 IRIF are unaffected by ZMYM2 loss. Experiments were performed in WT and ZMYM2 KO cells as in **Figure 4A**. Right panel: quantification RAP80 foci per cell after IR-treatment. Data represented as mean ± S.E.M. from > 100 cells, n = 2. (ns; not significant, One-way ANOVA test). Scale bar=20μm. **F** Interactions between BRCA1-A complex members RAP80, ABRA1 and BRCA1 are largely unaffected by ZMYM2 or ZMYM3 loss. Co-IP western blotting analysis of RAP80 with ABRA1 and BRCA1 was performed in WT, ZMYM2 KO and ZMYM3 KO U2OS cells. Input provides control for protein levels and IgG acts as a negative IP control.

Supplementary Figure S6

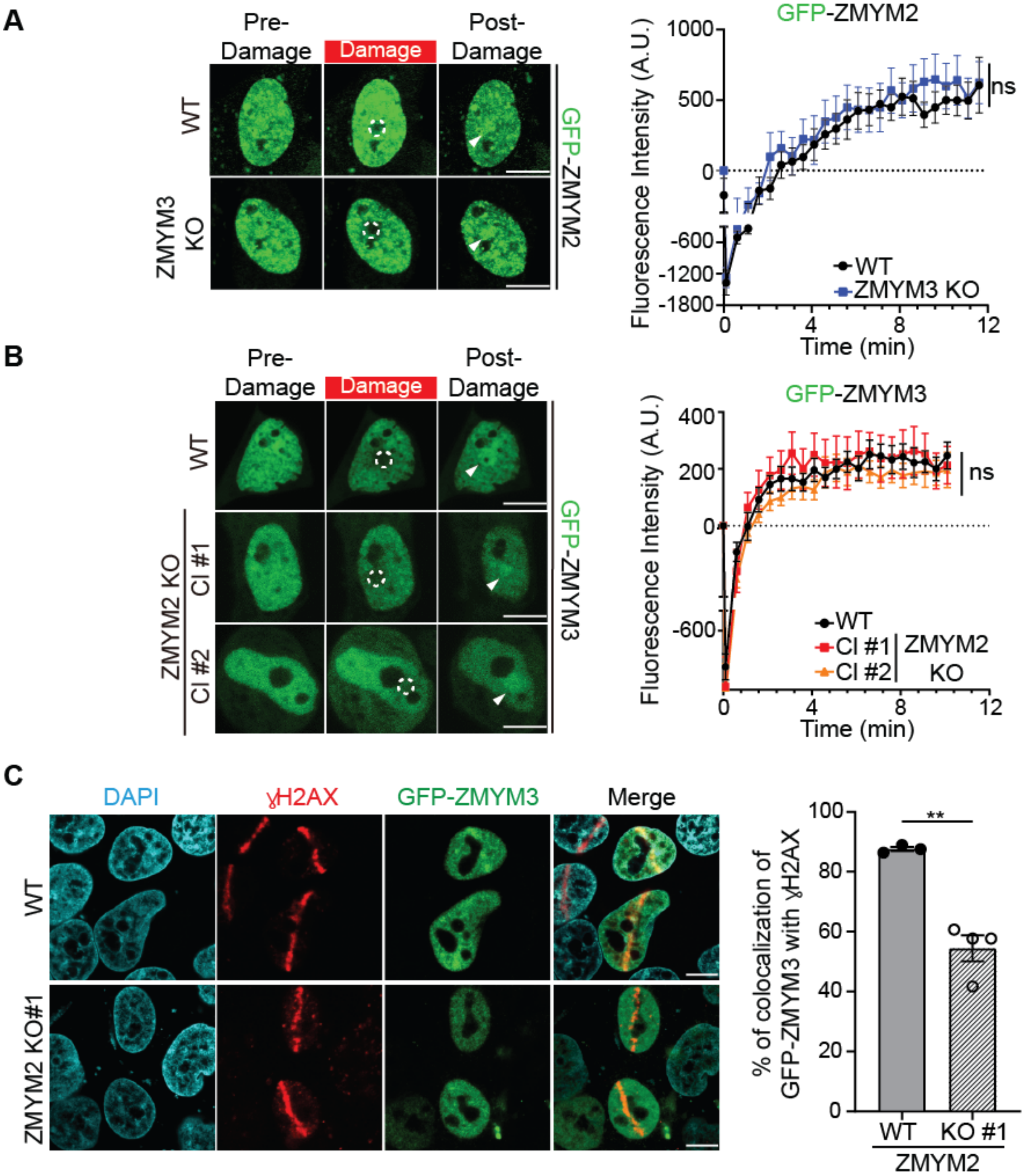

Supplementary Figure 6. ZMYM2 preferentially reinforces ZMYM3 accumulation at DNA lesions at late time points following DNA damage. **A** and **B** GFP-ZMYM2 and GFP-ZMYM3 rapidly associate with DNA damage independently from each other at early time points following DNA lesion production. GFP-ZMYM2 recruitment in ZMYM3 KO cells was analyzed in **A** and GFP-ZMYM3 recruitment in ZMYM2 KO cells was analyzed in **B**. Dotted white circles indicate damaged regions and white arrowheads point to post-damage regions. Data represent mean ± S.E.M., ≥ 9cells analyzed per condition. Scale bar=10μm. Right panels: Quantification of **A** and **B** (ns; not significant, Two-tailed unpaired t-test). **C** Recruitment of ZMYM3 to DNA damage is reduced in ZMYM2 KO cells 1 h post-lesion induction. U2OS WT and ZMYM2 KO cells expressing GFP-tagged ZMYM3 were analyzed by laser microirradiation 1-hour post-damage by IF analysis. γH2AX marks DNA damaged regions. Data represent mean ± S.E.M., ≥ 20 cells were analyzed per independent experiment, n = 3. scale bar=10μm. (**p=0.0013 two-tailed unpaired t-test).

Supplementary Figure S7

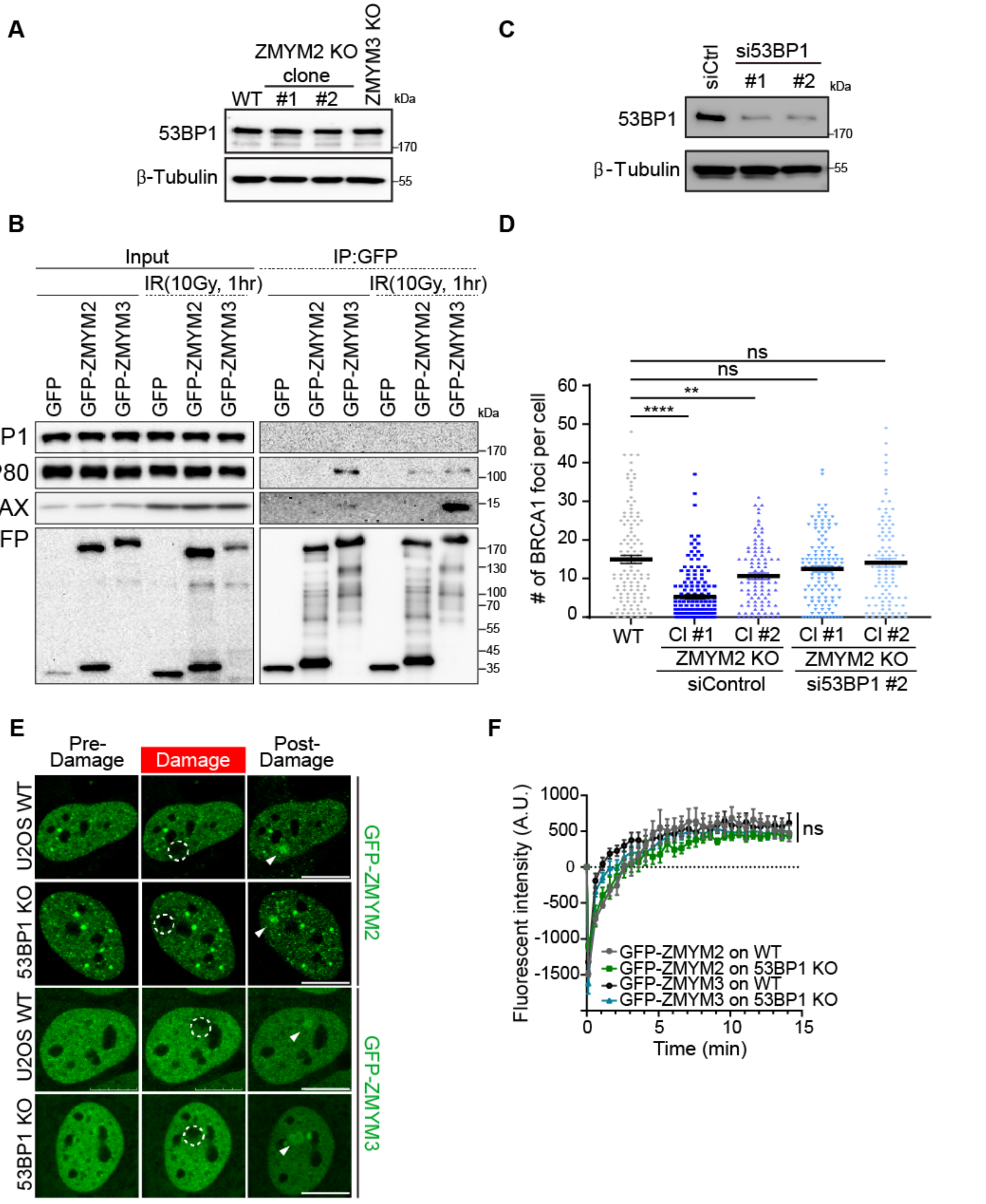

Supplementary Figure 7. 53BP1 loss rescues defective IR-induced BRCA1 foci formation in ZMYM2 KO U2OS cells. **A** 53BP1 protein levels are similar in WT, ZMYM2 or ZMYM3 KO cells. 53BP1 protein levels were confirmed in each cell line by western blotting. **B** 53BP1 does not associate with ZMYM2 and ZMYM3. Co-IP western analysis of GFP-tagged ZMYM2 or ZMYM3 with 53BP1, RAP80, and γH2AX was performed in HEK293T cells transiently expressing GFP, GFP-ZMYM2, or GFP-ZMYM3. γH2AX confirms DNA damage induction by IR. **C, D** Depletion of 53BP1 rescues BRCA1 IRIF defects that are present in ZMYM2 KO cells. Experiments were performed as in **Figure 7G** in WT and 2 independent ZMYM2 KO U2OS clones treated with either Ctrl or 53BP1 siRNAs. 53BP1 depletion by siRNAs was confirmed by western blotting as in **C** and quantification of BRCA1 foci following IR in WT and ZMYM2 KO cells with and without si53BP1 (siRNA #2) is shown in **D**. Data represent mean ± S.E.M. from > 120 cells, n = 3; (ns; not significant, ***$p<0.001$, One-way ANOVA test). **E** Loss of 53BP1 does not affect recruitment dynamics of ZMYM2 and ZMYM3 to laser damage. WT or 53BP1 KO U2OS cells transiently expressing GFP-ZMYM2 or GFP-ZMYM3 were damaged and analyzed 15 min post-damage by live-cell fluorescence confocal microscopy. Dotted white circles indicate damaged region and white arrowheads point to post-damage regions. Scale bar=10μm. **F** Quantification of DNA damage recruitment of ZMYM2 and ZMYM3 from **E**. Data represent mean ± S.E.M., n ≥ 9 cells were analyzed per condition. (ns; not significant, two-tailed unpaired t-test).
